# Response of different cotton genotypes to salt stress and re-watering

**DOI:** 10.1186/s12870-025-06534-6

**Published:** 2025-05-05

**Authors:** Kang Zhao, Tao Yang, Bo Pang, Honggang Wang, Zhining Yang, Weiwei Liang, Cun Rui, Wengwei Gao

**Affiliations:** 1 College of Agriculture, Xinjiang Agricultural University/Engineering Research Centre of Cotton, Ministry of Education, Urumqi, 830052 China; 2 Grass Industry Research Institute of Xinjiang Animal Science Academy, Urumqi, 830000 China; 3 Anyang Institute of Technology, Anyang, 455000 China

**Keywords:** Cotton, Salt stress, RNA-seq, Salt tolerance

## Abstract

**Background:**

Cotton is a vital economic crop and reserve material and a pioneer crop planted on saline-alkaline soil. Improving the tolerance of cotton to saline alkaline environments is particularly important.

**Results:**

Salt-tolerant and salt-sensitive cotton plants at the three-leaf stage were subjected to 200 mM NaCl stress treatment, thereafter, microstructural observations beside physiological and biochemical analyses were performed on cotton leaves at 0 h (CK), 48 h (NaCl) and re-watering (RW) for 48 h. Salt stress altered microstructural observations and physiological and biochemical in ST and SS (*p* < 0.05). After re-watering, ST recovered fully, while SS sustained permanent oxidative and structural damage, indicating distinct salt tolerance. Transcriptome analysis was performed on cotton leaves under salt stress and re-watering conditions. KEGG analysis revealed that the response of cotton to salt stress and its adaptation to re-watering may be related to major protein families such as photosynthesis (ko 00195), photosynthesis-antenna protein (ko 00196), plant hormone signal transduction (ko 04075), starch and sucrose metabolism (ko 00500), and porphyrin and chlorophyll metabolism (ko 00860). A gray coexpression module associated with cotton restoration under salt stress was enriched according to WGCNA.

**Conclusions:**

Salt stress did not only affect the physiological and biochemical levels of cotton but also induced structural changes in cells and tissues. Re-watering was relatively effective in stabilizing the physiological and biochemical parameters, as well as the leaf microstructure, of cotton plants under salt stress. WGCNA revealed enriched gray coexpression modules related to the recovery of cotton plants under salt stress, and screening of the pivotal genes in the gray module revealed five critical hubs, namely, *GH_A01G1528*, *GH_A08G2688*, *GH_D08G2683*, *GH_D01G1620* and *GH_A10G0617*. Overall, our findings can provide new insights into enhancing cotton salt tolerance and exploring salt tolerance genes in cotton,including screening cotton genetic resources using those potential responsive genes. This study provides a theoretical basis for further exploration of the molecular mechanism of cotton salt tolerance and genetic resources for breeding salt-tolerant cotton.

**Supplementary Information:**

The online version contains supplementary material available at 10.1186/s12870-025-06534-6.

## Background

Soil salinization seriously affects agricultural activities and food security and is an important ecological problem worldwide [1, 2]. Approximately 800,000 hectares of irrigated soil, or 40% of the total irrigated area, are affected by soil salinization worldwide [3]. With the increase in human activities and ecological deterioration, reclamation areas continue to expand, causing constant growth in soil salinization. It is estimated that by 2050, 50% of the world's agricultural land will experience varying degrees of soil salinization [4]. Moreover, secondary salinization in arid and semiarid regions of China tends to increase annually [5]. Xinjiang region has sparse precipitation, high evaporation, and special geology, which cause the accumulation of inorganic salts in the soil and make it prone to soil salinization [6]. Salt-alkaline soil is a general term for salinized soils, including saline soils, sodic soils, and saline sodic soils [7, 8]. Saline soil has a high content of neutral salts, including NaCl and Na_2_SO_4_. Accordingly, crop growth and development are negatively affected mainly by osmotic equilibrium, ion toxicity, nutrient absorption, synthesis, and respiration [9–13].

To adapt to salt stress, plants have developed various adaptive strategies [6, 13]. Salt-tolerant crop may increase leaf thickness [14] and root development [15] to improve water and nutrient absorption under salt stress. Compared with the normal ecotype of *Imperata cyclindrica* (L.) Raeuschel, which grows in salt-free environments, the solar pond ecotype under salt stress has thicker cuticles, more succulent leaves, more developed palisade tissues, and more sunken stomata [16]. In saline environments, plants may also regulate the composition and structure of cell walls to maintain cell stability and integrity [17]. High pH stress in salt-alkaline soil directly damages the absorption and transport of root ions. The efflux of K^+^ from roots causes K^+^-Na^+^ imbalance and a decrease in stomatal conductivity. Furthermore, as the concentration of soil salt-alkaline increased, the length, width, and pore size of the stomata decreased [18]. However, plants can redistribute ions and soluble compounds under salt stress to alleviate the adverse effects of salt on cellular function and metabolism [19]. For example, plants transport excessive Na^+^ from mesophyll cells to leaf veins, reducing Na^+^ toxicity in the cytoplasm and maintaining normal photosynthesis in leaves [20, 21]. Salt stress can induce excessive production of reactive oxygen species (ROS) in plants, leading to membrane damage [22]. Various enzymes with ROS scavenging abilities, such as catalase (CAT), superoxide dismutase (SOD), and peroxidase (POD), have been found in plants, all of which can enhance plant salt tolerance [23]. Plants may also synthesize antioxidants to counter damage induced by oxidative stress [24]. Melatonin, as a free radical scavenger and antioxidant, plays a critical role in improving the antioxidant system under salt stress [25]. Plants may change their patterns of gene expression under salt stress [26, 27], such as genes that directly protect plants from environmental stress and that regulate the expression of target genes in response to stress.

Cotton (*Gossypium hirsutum*), a member of the Mallow family, is a pioneer crop in the development and utilization of salt-alkaline land [28]. It is a major economic crop in China, holding a prominent position in national politics, economy, culture, and social life [29]. High salt stress affects chlorophyll synthesis, resulting in slow and abnormal seedling growth and difficulty in spreading two cotyledons [30]. Cotton plants are sensitive to salt stress during the two-leaf and trefoil stages, during which flower bud differentiation takes place [31]. Usually, cotton plants exhibit stress symptoms such as leaf margin carving, leaf color darkening, cotyledon shedding, and plant wilt under salt stress. The increase in osmotic pressure in the soil solution leads to physiological drought and ion accumulation and hinders the absorption of nutrients and water [32]. Studies of salt tolerance in cotton have shown that important membrane receptors involved in the biosynthesis and signal transduction of calc-dependent protein kinases, mitogen-activated protein kinases and hormones (abscisic acid and ethylene), as well as transporters and pathcode-coding genes, are upregulated [33]. By performing different salt-alkaline treatments on upland cotton, the specific alkaline resistance candidate gene *GhA12G2168* (cystathionine γ-synthase gene *ChMGL11*) was identified [34]. Melatonin can enhance cotton salt tolerance by regulating various mechanisms, such as antioxidant systems, plant hormones, transcription factors, phospholipid metabolism, and signaling molecules [35]. In cotton-bacteria-salt interactions, symbiotic metabolites (e.g., inositol, galactose) are converted into raffinose, enhancing osmoregulation and scavenging free radicals to mitigate salt stress [36]. These findings provide new ideas for breeding salt-tolerant cotton and are expected to be further applied in related fields. Furthermore, salt stress critically impairs cotton root development, exacerbating water and nutrient acquisition challenges, ultimately diminishing yield and fiber quality. In our previous investigation conducted in natural saline-alkali soils of Xinjiang, we systematically evaluated 319 upland cotton accessions across four developmental stages (seedling emergence, flowering-boll, and boll-opening phases), analyzing agronomic traits, physiological parameters, yield components, and fiber quality metrics. Our results corroborate the aforementioned literature. Notably, we identified substantial divergence in salt tolerance between two germplasms: The Yangtze River Valley- 52–128 and The Yellow River Valley-Jing Si Mian, highlighting distinct regional adaptation mechanisms in cotton genetic resources.

For plants under salt stress, re-watering can effectively restore their phenotype, physiology, and gene expression, alleviating the influence of salinity on plants. However, there is limited research on how re-watering affects plant responses to salt stress [37]. A deep understanding of the physiological and molecular mechanisms of salt stress and re-watering is conducive to protecting and efficiently utilizing water resources [38]. In addition, because of its high efficiency in mining function-related genes in coexpression modules, weighted gene coexpression network analysis (WGCNA) has been extensively applied to mine corresponding candidate genes in crops [39]. This study investigated the response of cotton to salt stress and its adaptability after re-watering through morphology, physiology, and microstructure analyses. Moreover, cotton salt tolerance genes were identified through RNA-seq technology.

## Materials and methods

### Experimental design

This study used two cotton genotypes with significant differences in salt tolerance, namely, The Yangtze River Valley- 52–128 (Salt-Sensitive genotype, SS) and The Yellow River Valley-Jing Si Mian (Salt-Tolerant genotype, ST). Cotton seeds were collected from the experimental base of Xinjiang Agricultural University, Shawan County, Xinjiang Uygur Autonomous Region. Natural salt-tolerant soil experiments were conducted in Shawan, China, from 2018 to 2019 (Table S1). Du et al. [40] verified the differences in salt tolerance between these two genotypes during the seedling stage.The seeds were disinfected with 5% sodium hypochlorite (NaClO) for 20 min, rinsed three times with sterile water, sown on filter paper in germination boxes (12.4 cm × 17.5 cm) pre-moistened with 14 mL ddH₂O, and incubated under controlled conditions (day/night temperatures of 26 °C/18 °C, relative humidity 65%, 8 h/16 h light/dark cycle, 276 µmol s^−1^ m^−2^ light intensity; stored in artificial climate boxes).. After the cotyledons were fully unfolded, the seedlings were transferred to a 1/2 Hoagland nutrient solution until the trefoil stage. Cotton seedlings at the trefoil stage were cultivated in a 200 mM NaCl solution for 48 h and then cultured in 1/2 Hoagland nutrient solution for 48 h (re-watering, RW). Samples were taken at 0 h, 48h, and RW48h. The vessels used to culture the plants were 500 ml tinfoil wrapped glass conical flasks. There were 100 plants with three biological replicates. A total of 18 sets of samples (3 time points × 2 species × 3 biological replicates) were collected for subsequent Physiological Measurement, Morphological observation and RNA-seq.

### Physiological measurement

The maximum functional leaves of cotton plants under different treatments were measured using a portable chlorophyll analyzer (SPAD- 502Plus, USA Spectrum). The malondialdehyde content, superoxide dismutase activity, and peroxidase activity were measured using thiobarbituric acid colorimetry [41], the nitrogen blue tetrazole photoreduction method [42], and the guaiacol hydrogen peroxide method, respectively [43].

### Microstructural observations

Microstructural observations were made with reference to Yuan et al. [44]. Leaves from the different treatment groups were cut vertically from veins and placed in FAA fixation solutions separately. Pruning, dehydration, embedding, slicing, and staining should be carried out when the leaves are in good fixed condition. Finally, the sample was sealed through a microscope and stained with saffron rapid green staining solution. Images were collected and scanned using a panoramic slicing scanner (models: PANNORAMIC DESK/MIDI/250/1000 and CaseViewer2.2) produced by 3DHISTECH (Hungary). Image-Pro Plus 6.0 produced by Media Cybertics (USA) was used to analyze these images. The thicknesses of each slice at five locations, namely, the upper epidermis, palisade tissue, sponge tissue, and lower epidermis, were measured and averaged.

### RNA extraction and cDNA library construction

At 0 h and 48 h of NaCl treatment and at 48 h of RW treatment, the leaves of the ST and SS plants were collected and immediately frozen in liquid nitrogen. Then, all the samples were stored at − 80 °C for RNA-Seq analysis. Total RNA was extracted according to the product manual of Tiangen's polysaccharide and polyphenol total RNA extraction kit. RNA degradation and contamination were detected by 1% agarose gel electrophoresis. A Nanodrop 2000 (Thermo Fisher Nanodrop 2000, Shanghai, China) was used for concentration detection. An Agilent 2100 (Platinum Elmer LabChip GX, Beijing, China) was used to calibrate the RNA integrity. The RNA concentration was calibrated based on qubit quantitation (Life Technologies, Beijing, China). The samples with OD260/280 values ranging from 1.8 to 2.2 and OD260/230 g ranging from 1.8 to 2.2 were qualified. Qualified RNAs were sent to Beijing Biomarker Biological Company for cDNA library construction. Libraries were constructed using the NEBNext Ultra II DNA Library Prep Kit and sequenced on an Illumina NovaSeq 6000 system (paired-end 150 bp mode) with a minimum of 6G raw data per sample.

### Data and Differentially Expressed Gene (DEG) analysis

Original readings were obtained using Illumina. First, fastp software [45] was used for data filtering and quality control (Raw reads were processed by removing adapters and filtering low-quality sequences, Q-score ≤ 10 in > 50% bases or Ns > 10%). Clean reads were obtained while calculating the Q30 and GC content. Then, HISAT2 software was used to map the clean reads to the cotton reference genome Gossypium_Hirsutum.TM_1_V2.1.genome.fa [46]. StringTie software [47] was used for quantification based on fragments per kilobase of transcript per million fragments mapped (FPKM) to estimate the gene expression level of each transcript [48]. Using the edge R package [49], genes with a fold change ≥ 2 and an FDR < 0.01 were considered DEGs (differentially expressed genes) [50]. The false discovery rate (FDR) was calculated by adjusting the p value multiple times [51].

### WGCNA construction

Through the BMKCloud cloud platform (www.biocloud.net), a cotton salt tolerance coexpression network was established for 23,707 DEGs produced by ST and SS under salt stress and re-watering. In the scale-free weighted gene network, nodes corresponded to DEGs, and edges were determined by similar expression profiles of paired genes calculated through Pearson correlation. Therefore, an optimal soft threshold β = 26 was chosen to construct a network based on an adjacency matrix (*R*^2^ > 0.8). The topological overlap matrix was calculated through the adjacency matrix, and the dynamic tree cutting algorithm was used to segment the modules according to default parameter settings. A grouping matrix of 18 samples was used as a trait for specificity module identification. Cytoscape 3.9.1 software was used to visualize the coexpression networks.

### qRT‒PCR validation of the transcriptome

To verify the Illumina sequencing results, RNA was extracted from SS and ST plants under salt stress conditions at 0 h, 48h, and RW48h. Ten DEGs were randomly selected, and conserved regions of the sequence were identified via BLASTX and ORF finders in the NCBI. An online website (https://www.primer3plus.com) was utilized to design the qRT‒PCR primers (Table S2). Based on previous research [52], with *GhUBQ7* as the internal reference, a two-step program was used to detect changes in gene expression using the ABI7500 Fast System (USA ABI Company). Each reaction was repeated three times.Finally, the relative expression of Ten DEGs was calculated using the 2-ΔΔCt method [53].

### Data analysis

The statistical analysis was performed using IBM SPSS Statistics 27. A two-way analysis of variance (ANOVA) was conducted to compare the differences in physiological and biochemical levels and leaf microstructure between two genotypes under different treatments, followed by post-hoc multiple comparisons using the Least Significant Difference (LSD) method. The sequencing results were analyzed through the BMKCloud cloud platform (www.biocloud. net).

## Results

### Phenotypic observation of cotton seedlings under salt stress and re-watering

**Fig. 1:**
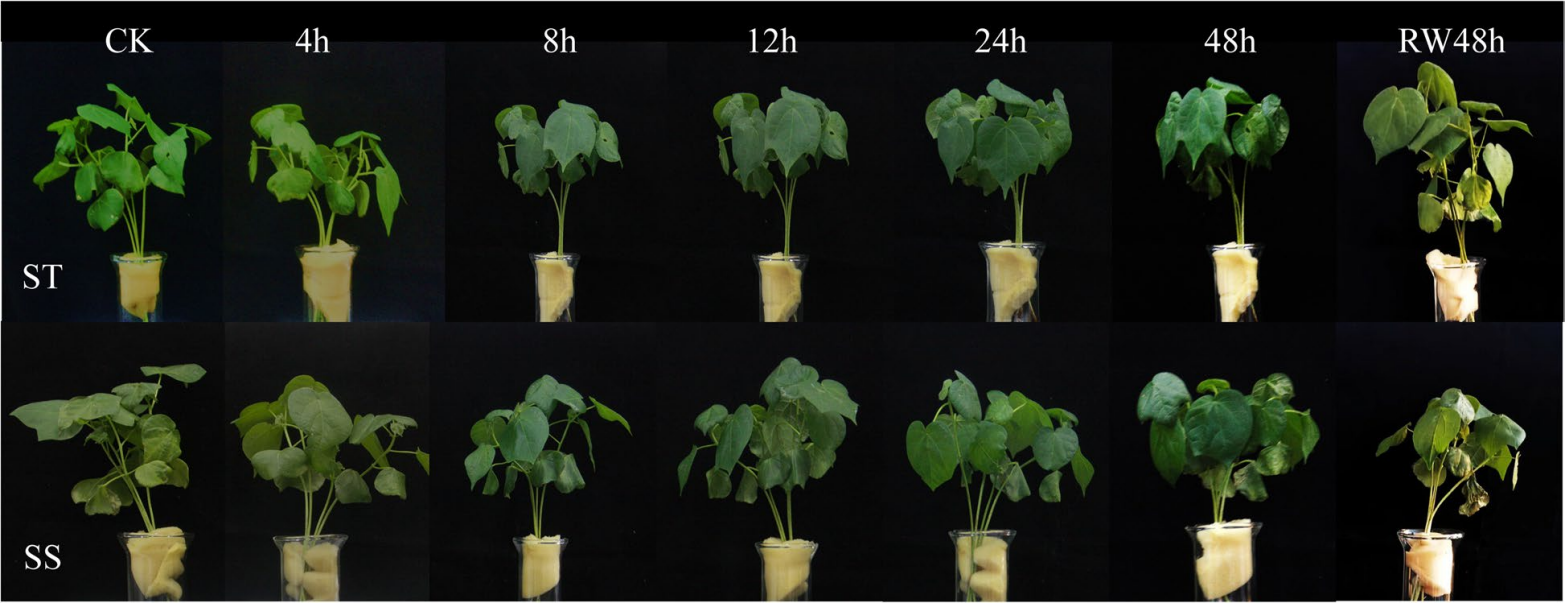
Growth of cotton plants under salt stress conditions at different time points. CK: Control group; RW48 h:re-watering 48 h; SS: Salt-Sensitive genotype; ST: Salt-Tolerant genotype

Cotton plants at the trefoil stage were treated with 200 mM NaCl solution. The growth of cotton plants under salt stress was observed at different time points (Fig. 1). Under NaCl stress, the leaves and stems of the ST and SS plants gradually softened, and the cotyledons wilted severely at 48 h. Compared with those of the ST plants, the leaves and stems of the SS plants changed significantly, their cotyledons withered and fell at 48 h, and their true leaf margins were slightly charred. After 48 h of re-watering, ST growth was relieved, and the true SS leaves wilted.

### Physiological responses of cotton seedlings to salt stress and re-watering

**Fig. 2:**
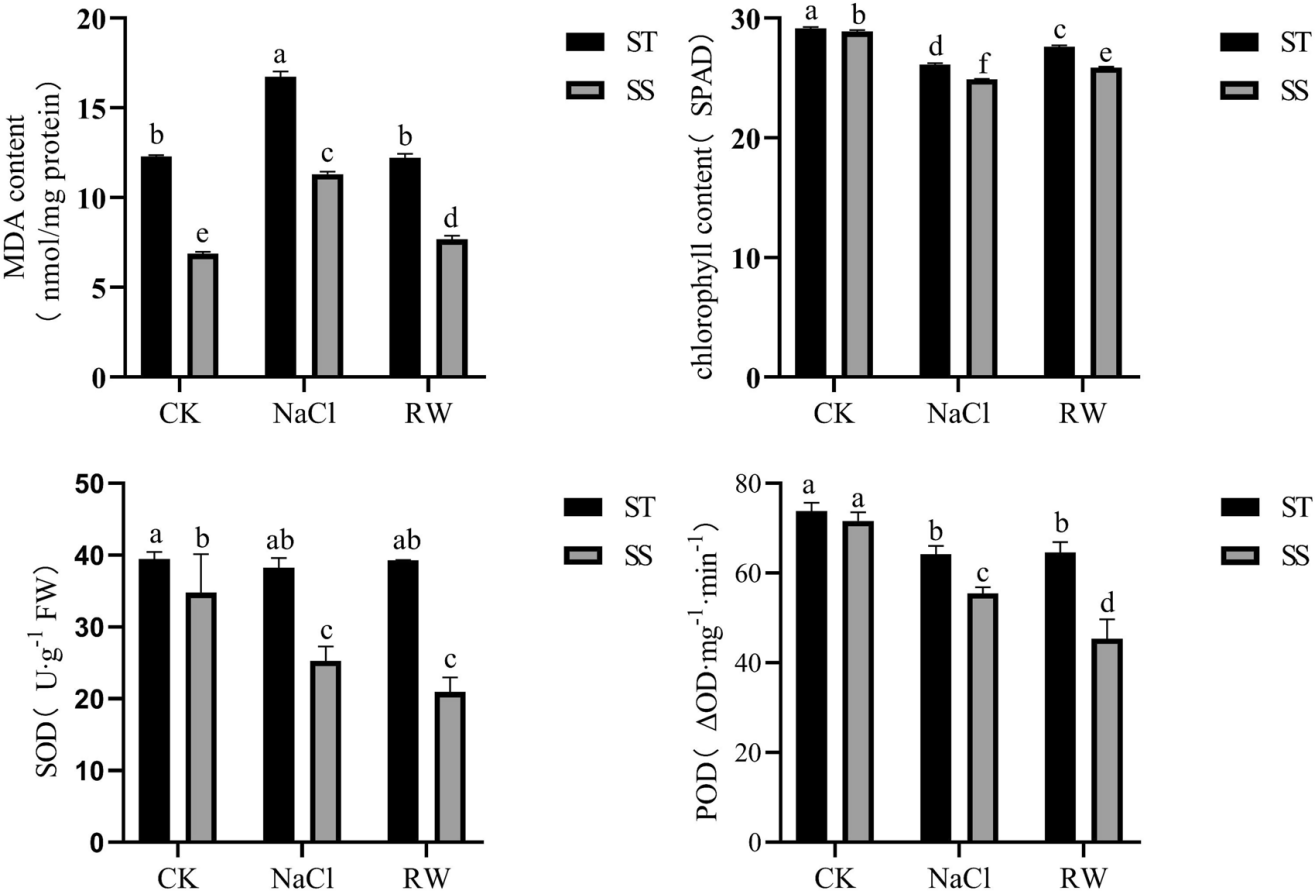
Physiological response of cotton seedlings to salt stress and re-watering. The same letters indicate no significant differences between treatments (*P* < 0.05, LSD method); The results were presented as the mean ± SD (standard deviation) of the three measurements. CK:Control group; NaCl: NaCl 48 h; RW: re-watering 48 hSS:Salt-Sensitive genotype; ST:Salt-Tolerant genotype

Salt stress can disrupt the ion balance within plant cells. The generated ion toxins, osmotic pressure, and reactive oxygen subsequently affect the growth and development of cotton. As shown in Fig. 2, the malondialdehyde concentrations of the ST and SS plants significantly increased, and the chlorophyll content, superoxide dismutase activity and peroxidase activity decreased under NaCl treatment for 48 h, indicating that salt stress affected the normal growth and development of the cotton plants. The malondialdehyde concentrations in the ST and SS treatments increased by 36.07% and 64.61%, respectively, compared with that in the CK treatment, and after 48 h of re-watering, the malondialdehyde concentration in the ST treatment did not change, while the malondialdehyde concentration in the SS treatment increased by 11.54% compared with that in the CK treatment. This indicated that salt stress significantly affected the lipid peroxidation level and that re-watering had a greater mitigating effect on ST than on SS. The relative chlorophyll contents of the ST and SS plants were reduced by 10.29% and 13.89%, respectively, compared with those of the CK plants under salt stress and by 5.23% and 10.36%, respectively, compared with those of the CK plants after re-watering. This indicates that salt stress significantly inhibited chlorophyll synthesis in cotton and that re-watering also significantly improved chlorophyll synthesis in both ST and SS. Compared with those in the CK treatment, the superoxide dismutase activity and peroxidase activity in the ST treatment decreased by 3.06% and 12.98%, respectively, and after re-watering, they decreased by 0.36% and 12.51%, respectively. The superoxide dismutase activity and peroxidase activity in SS were significantly reduced by 27.5% and 13.89%, respectively, compared with those in CK and were reduced by 5.23% and 10.36%, respectively, compared with those in CK after re-watering under salt stress. Compared with those in the CK treatment, the stress in the CK treatment significantly decreased by 27.31% and 22.46%, respectively, and after re-watering, the stress significantly decreased by 39.79% and 36.67%, respectively. This indicated that the ability of ST to clear reactive oxygen species under salt stress was greater than that of SS and did not improve re-watering.

### Microstructures of cotton leaves under salt stress and re-watering

**Fig. 3:**
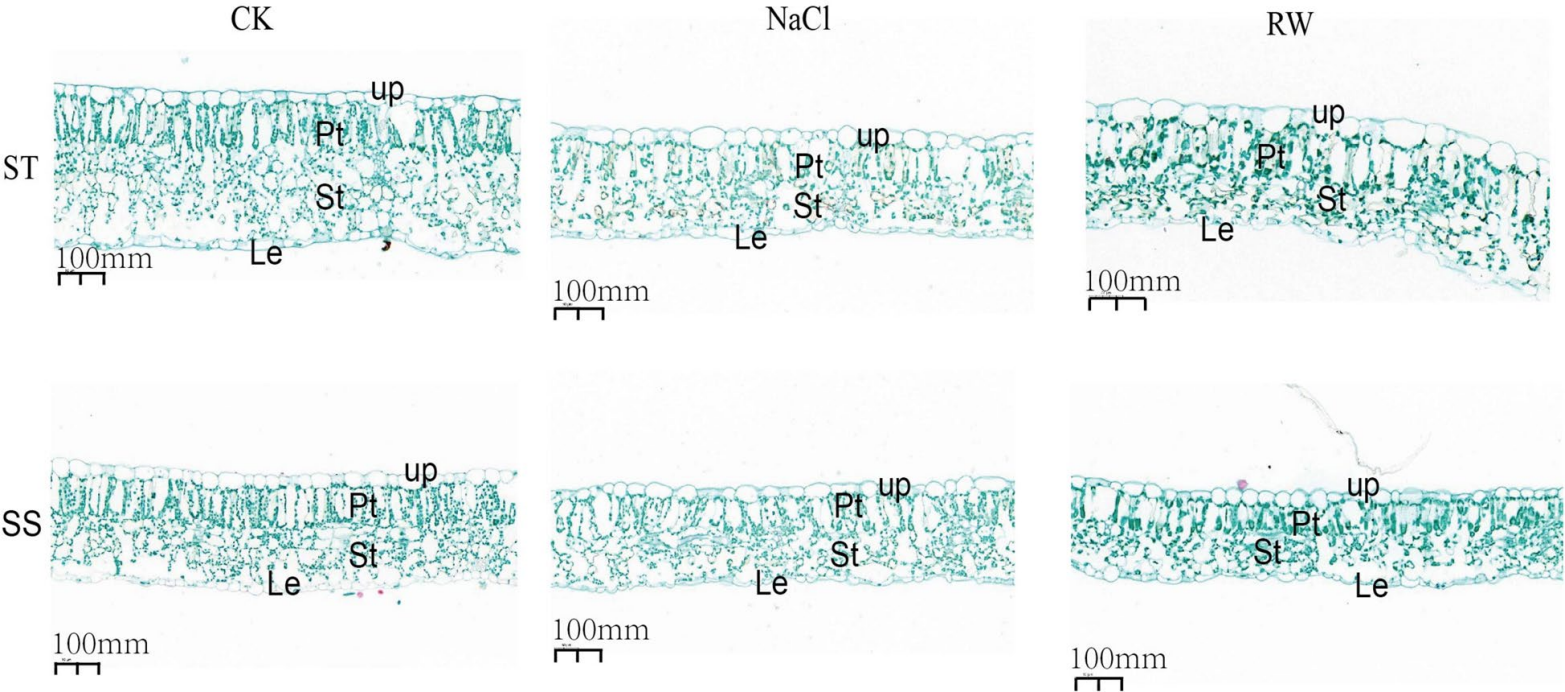
Microstructure of cotton leaves under salt stress and re-watering. Upper, upper epidermis; Pt, palisade issue; St, spongy issue; Le, lower epidermis. SS;CK:Control group; NaCl: NaCl 48 h; RW: re-watering 48 h; CK:Control group; NaCl: NaCl 48 h; RW: re-watering 48 hSS:Salt-Sensitive genotype; ST:Salt-Tolerant genotype

Salt stress not only affects the physiological and metabolic processes of plants but also causes changes in plant morphology, anatomical structure, and other aspects. According to the microstructure of the cotton leaves under salt stress and re-watering (Fig. 3), the ST and SS seedling slices were deeply stained, the microstructure was tightly arranged, the bulliform cells were shaped like single-layer irregular quadrilaterals and closely arranged under normal conditions, the bulliform cells were ovoid, the arrangement of palisade and sponge tissue cells was relatively loose, and the leaves were softened. After re-watering, the bulliform cells had a long egg shape, and the leaves were relatively flat, gradually returning to normal growth.

Analysis of the leaf epidermal structure revealed that under normal conditions, there was no significant difference in the epidermal structure of the leaves between the ST and SS cotton plants (Table S3). Under NaCl stress, the leaf thickness, palisade tissue thickness, and sponge tissue thickness of the ST and SS plants all decreased, and they relatively recovered after re-watering. After NaCl treatment, compared with those of the control, the leaf thickness, palisade tissue thickness, and sponge tissue thickness of the ST seedlings decreased by 16.17%, 13.92%, and 18.46%, respectively, without any significant difference; those of the SS decreased by 32.43%, 30.13%, and 41.10%, respectively. After re-watering, the leaf thickness, palisade tissue thickness, spongy tissue thickness and upper epidermis thickness of the ST cotton seedlings increased by 0.93%, 0.09%, 3.41% and 2.28%, respectively, and the leaf thickness, palisade tissue thickness and spongy tissue thickness of the SS plants significantly decreased by 28.60%, 28.71% and 32.53%, respectively, compared to those of the control plants.

### Transcriptome sequencing analysis of cotton seedlings under salt stress and re-watering

To explore key genes related to salt tolerance in cotton, this study sampled two cotton genotypes whose salt tolerance significantly differed under 200 mM NaCl stress at 0 h, 48 h, and 48 h of re-watering. Each treatment had three biological replicates. The 18 collected samples (3 time points × 2 varieties × 3 biological replicates) were used to construct 18 RNA-seq libraries, and Illumina HiSeq 4000 sequencing and analysis were performed. A total of 127.58 Gb of clean data were obtained, with a Q30 percentage of no less than 91.70% for all the samples. Sequence alignment was performed between the total reads of each sample and the reference genome (TM- 1) of upland cotton. According to the alignment results, the alignment efficiency between the reads of each sample and the reference genome was between 94.06% and 96.60%, and the GC content of each sample exceeded 43.47%. These results demonstrated that the RNA-seq data in this study were of high quality and could be further analyzed (Table S4).

Based on the comparison results, 11,125 genes were discovered, of which 6,974 were functionally annotated. The normalized FPKM value was used to measure the expression level of each gene, and the Pearson correlation coefficient (PCC) was used to detect the correlation between all samples. The overall correlation between the three biological replicates of the two varieties at the same growth stage was high. Moreover, sample clustering exhibited good correlation between biological replicates in the same environment (Fig. S1A). To further confirm the correlation between ST and SS at different stages under salt stress and re-watering, principal component analysis was performed on the genes expressed as described above (Fig. S1B). These results indicated that the two cotton varieties with significant differences in salt tolerance have apparently diverse expression patterns in different environments. SS and ST exhibited consistency in three biological replicates in the same environment, indirectly verifying the reliability of the transcriptome data.

**Fig. 4:**
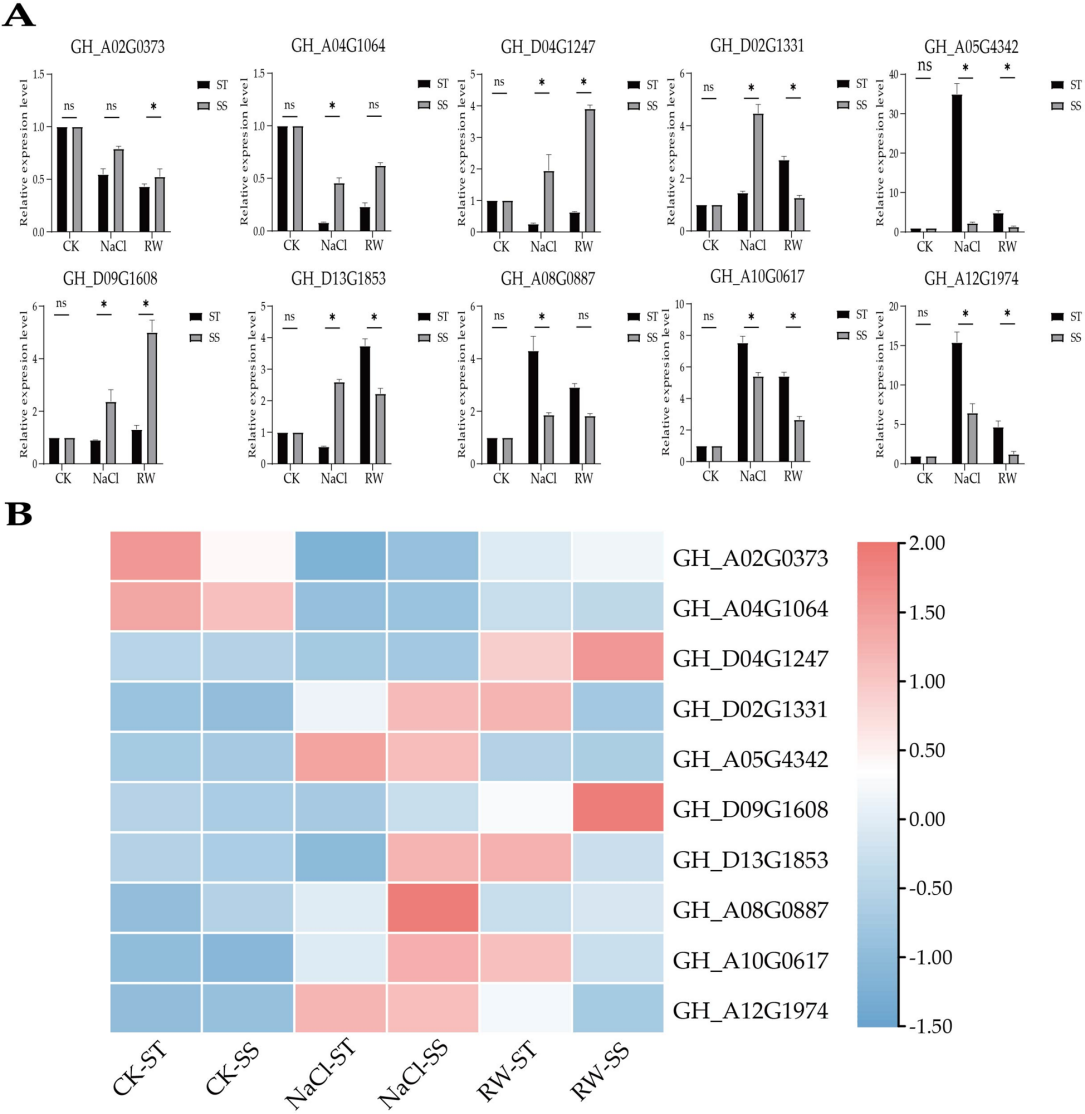
qRT‒PCR validation of the RNA-Seq results. **A** qRT‒PCR results for ten genes. The relevant significance level (p value) has been added above each histogram. Ns indicates no significant impact at the *p* < 0.05 level, The results were presented as the mean ± SD (standard deviation) of the three measurements. while * indicates a significant impact at *p* < 0.05. The black strip represents ST; the gray bar represents SS. **B** RNA-seq heatmap of ten genes. SS; CK:Control group; NaCl: NaCl 48 h; RW: re-watering 48 h; CK: Control group; NaCl: NaCl 48 h; RW: re-watering 48 hSS: Salt-Sensitive genotype; ST: Salt-Tolerant genotype

**Fig. 5:**
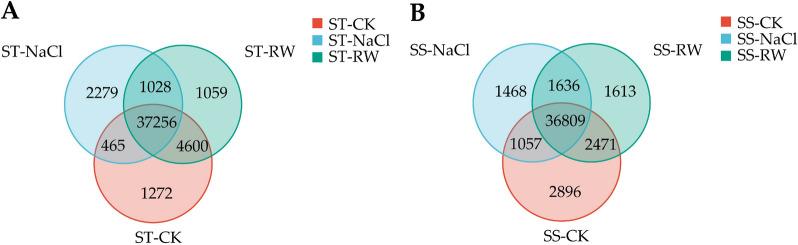
**VN A** Venn diagram of gene expression in ST leaves*;* **B** Venn diagram of gene expression in SS leaves. SS;CK: Control group; NaCl: NaCl 48 h; RW: re-watering 48 h; CK: Control group; NaCl: NaCl 48 h; RW: re-watering 48 hSS: Salt-Sensitive genotype; ST: Salt-Tolerant genotype

To confirm the reliability of the RNA-Seq results in this study, ten genes were randomly selected for qRT‒PCR (Table S2). The results were consistent with the expression trend of the transcriptome expression profile, proving that the transcriptome data were reliable for subsequent analysis (Fig. 4). In SS, 43,233, 40,970, and 42,529 genes were expressed in sequence, of which 36,890 genes were coexpressed. There were 43,593, 41,028, and 43,943 genes expressed in STs, respectively, of which 37,256 genes were coexpressed (Fig. 5).

### `Analysis of DEG expression between the two cotton genotypes

**Fig. 6:**
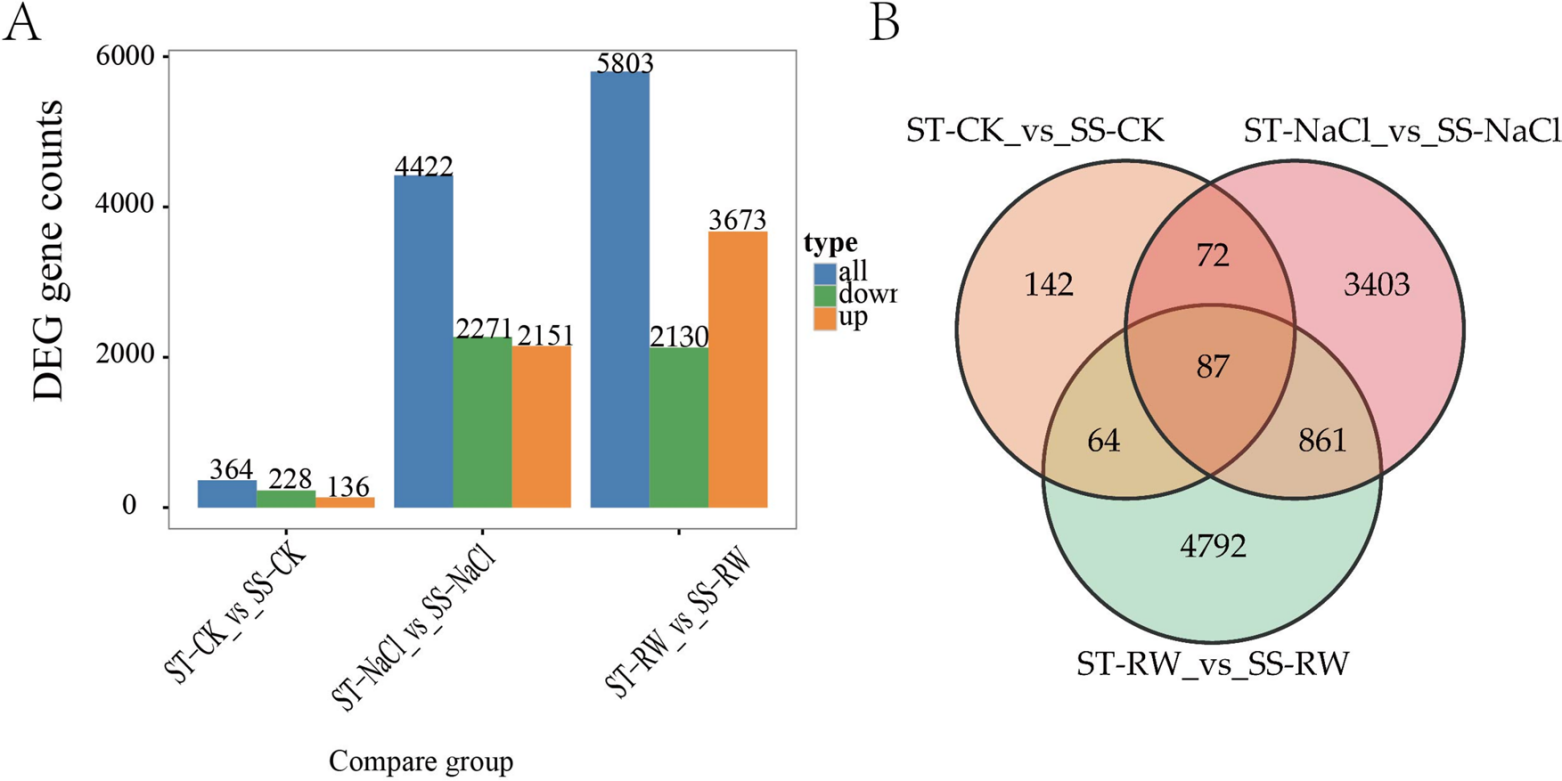
GO analysis of DEGs between genotypes subjected to the same treatment. GO analysis of DEGs in both genotypes under Control group (ST-CK vs. SS-CK); GO analysis of DEGs in both genotypes under NaCl stress (ST-NaCl vs. SS-NaCl); GO analysis of DEGs in both genotypes under re-watering (ST-RW vs. SS-RW);

Comparative analysis of differentially expressed genes (DEGs) between the two genotypes against the Gene Ontology (GO) database revealed distinct enrichment patterns (Fig. 6). In the biological process category, DEGs were predominantly enriched in metabolic processes (e.g., carbohydrate and lipid metabolism), cellular processes (e.g., ion homeostasis and cell cycle regulation), and single-organism processes (e.g., stress response). Within cellular components, DEGs were primarily associated with cell and cell part structures, including membranes and organelles. Molecular function analysis highlighted enrichment in binding (e.g., nucleic acid and protein binding) and catalytic activities (e.g., oxidoreductases and transferases). Notably, a substantial number of genes were downregulated under normal conditions, whereas re-watering induced the upregulation of multiple stress-responsive genes, indicating transcriptional reprogramming during recovery.

**Fig. 7:**
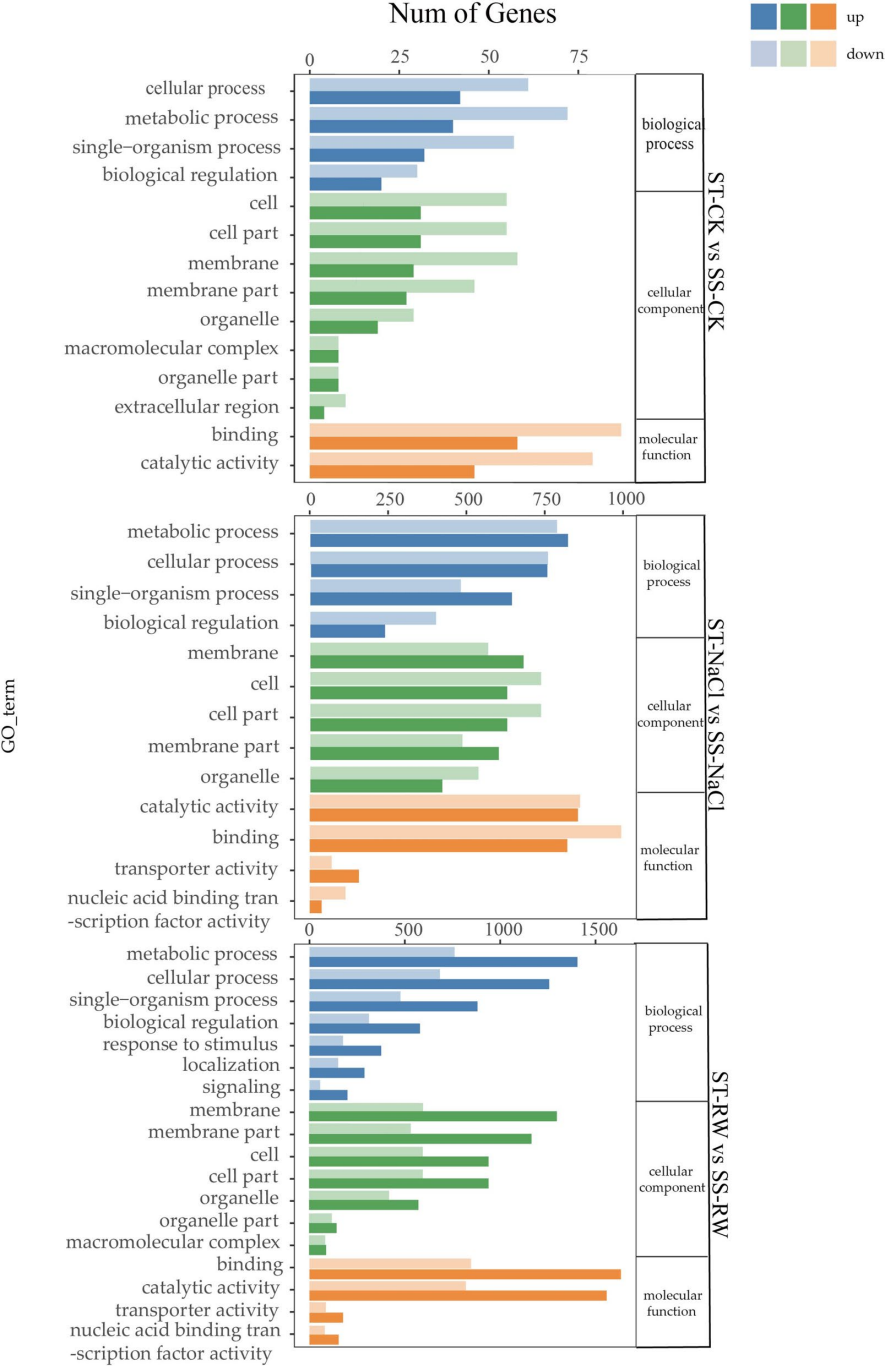
KEGG analysis of DEGs between genotypes subjected to the same treatment. **A** KEGG analysis of DEGs under Control group (ST-CK vs. SS-CK); **B** KEGG analysis of DEGs under salt stress (ST-NaCl vs. SS-NaCl); **C **KEGG analysis of DEGs under re-watering (ST-RW vs. SS-RW)

Kyoto Encyclopedia of Genes and Genomes (KEGG) enrichment analysis of DEGs identified genotype-specific pathways (Fig. 7). In salt-tolerant (ST) plants, DEGs were significantly enriched in the following pathways:Photosynthesis-antenna proteins (ko00196), involving light-harvesting complex genes (e.g., Lhcb1 and Lhcb4);Carbon fixation in photosynthetic organisms (ko00710), including key enzymes such as ribulose bisphosphate carboxylase (RuBisCO);Vitamin B6 metabolism (ko00750), encompassing pyridoxal kinase and phosphatase genes.

### Expression analysis of DEGs under salt stress and re-watering

**Fig. 8:**
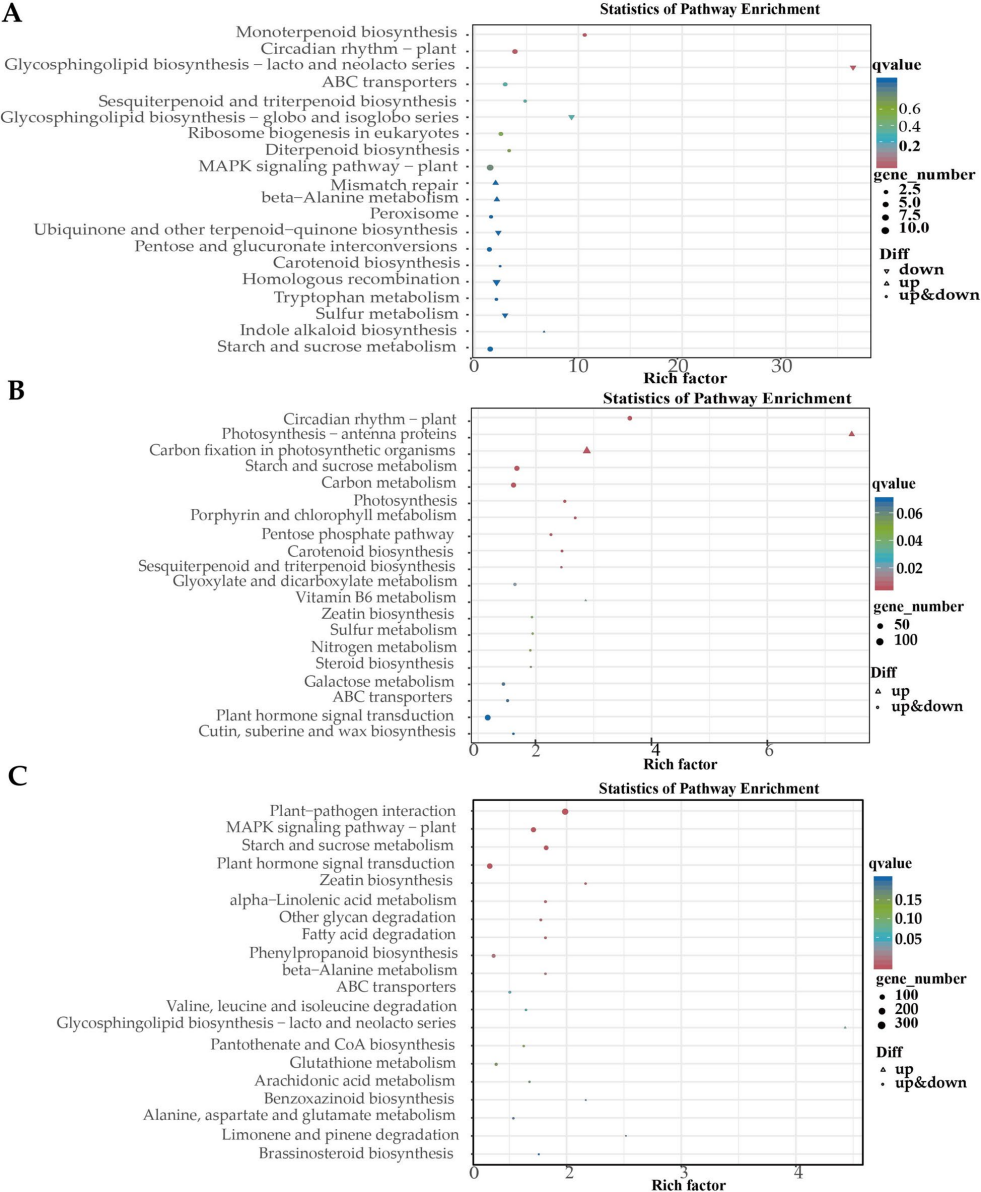
DEGs between treatments. **A** Histogram of DEGs between treatments; **B** Wayne plot of DEGs between treatments

A fold change ≥ 2 and FDR < 0.01 were adopted as screening criteria to obtain DEGs under salt stress and re-watering for further analysis of the differential mechanisms (Fig. 8). Under salt stress, 15,780 genes were screened in the ST, of which 7,996 DEGs were upregulated, and 7,784 DEGs were downregulated. Under re-watering, 2,454 genes were screened, including 659 upregulated genes and 1,795 downregulated genes. For SS under salt stress, 14,156 genes were screened. Among them, 6,925 DEGs were upregulated, and 7,231 DEGs were downregulated. Under re-watering, 9,390 genes were screened in SS, of which 4,666 DEGs were upregulated and 4,724 DEGs were downregulated. Under salt stress, the number of DEGs screened in ST was greater than that in SS. However, fewer DEGs were produced after re-watering in the ST treatment than in the SS treatment, which may be related to the greater recovery of the STs after re-watering.

**Fig. 9:**
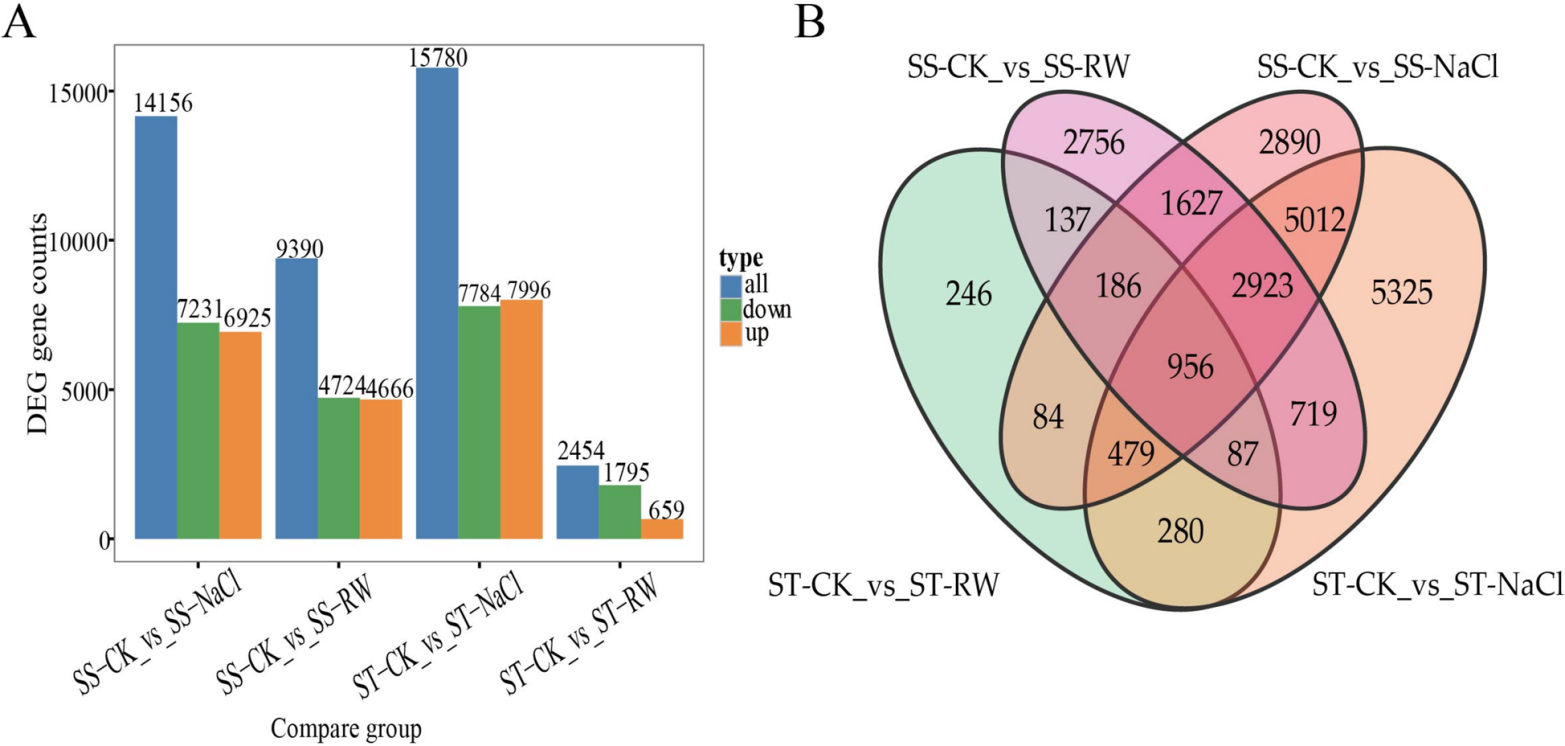
GO analysis of DEGs between treatments. GO analysis of DEGs in ST under salt stress (ST-CK vs. ST NaCl); GO analysis of DEGs in ST under re-watering (ST-CK vs. ST-RW); GO analysis of DEGs in SS under salt stress (SS-CK vs. SS-NaCl); GO analysis of DEGs in SS under re-watering (SS-CK vs. SS-RW)

The DEGs of the two genotypes under salt stress and re-watering were compared via the GO database (Fig. 9). In terms of biological processes, DEGs were mainly enriched in metabolic processes, cellular processes, and single organism processes. For cellular components, DEGs were mainly involved in cells, cellular components, and membranes. Regarding molecular functions, DEGs were mainly enriched in binding and catalytic activities.

**Fig. 10:**
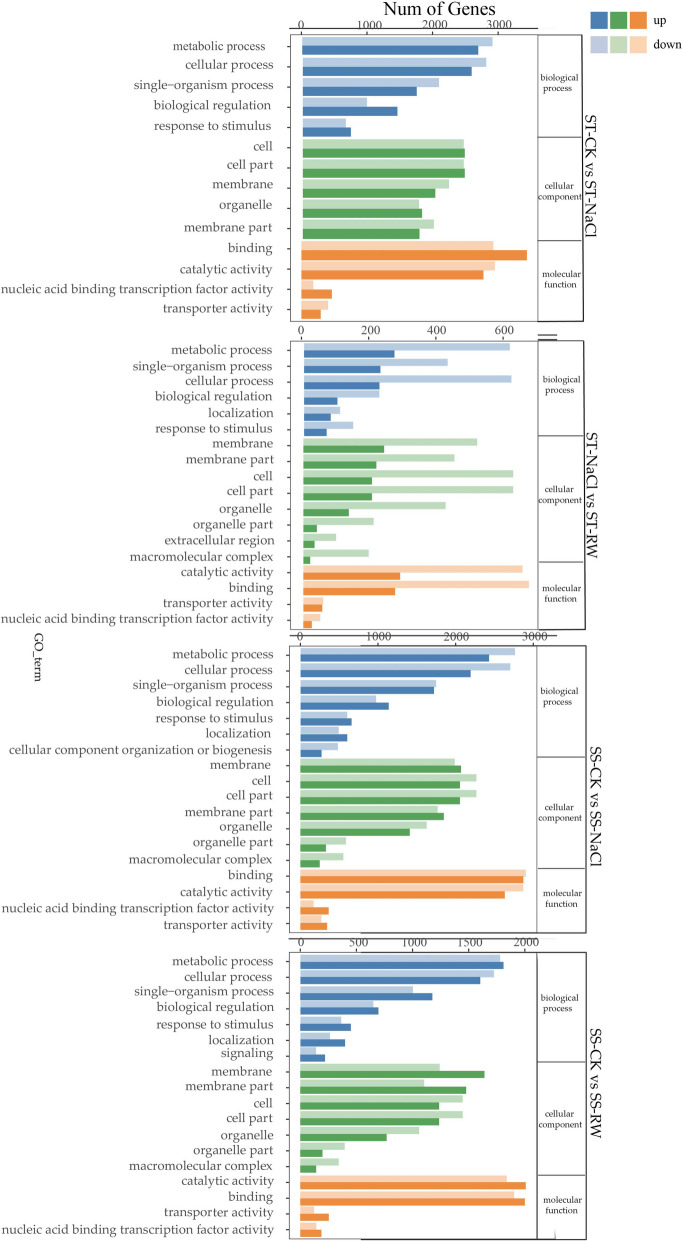
KEGG analysis of DEGs between treatments. **A** KEGG analysis of DEGs in ST under salt stress (ST-CK vs. ST NaCl); **B** KEGG analysis of DEGs in ST under re-watering (ST-CK vs. ST-RW); **C** KEGG analysis of DEGs in SS under salt stress (SS-CK vs. SS-NaCl); **D** KEGG analysis of DEGs in SS under re-watering (SS-CK vs. SS-RW)

DEGs annotated by KEGG were classified into multiple categories (Fig. 10). The response of cotton to salt stress and its adaptation to re-watering may be related to interactions such as photosynthesis (ko 00195), photosynthesis-antenna protein (ko 00196), plant hormone signal transduction (ko 04075), starch and sucrose metabolism (ko 00500), and porphyrin and chlorophyll metabolism (ko 00860). Additionally, STs respond to salt stress through pathways such as circadian rhythm-plant (ko 04712), carbon fixation (ko 00710) and carbon metabolism (ko 01200) pathways in photosynthetic organisms. The adaptability of STs to re-watering was regulated through pathways such as glyceride metabolism (ko 00561), the reciprocal transformation of pentose and glucuronate (ko 00040), circadian rhythm-plant (ko 04712), and phenylpropane biosynthesis (ko 00940). SSs resist salt stress through pathways such as fatty acid degradation (ko 00071); degradation of valine, leucine, and isoleucine (ko 00280); biosynthesis of brassinosteroid (ko 00905); and sphingolipid metabolism (ko 00600). The damage caused by salt stress was alleviated through plant‒pathogen interactions (ko 04626), the MAPK signaling pathway (ko 04016), and α-linolenic acid metabolism (ko 00592).

### Effects of salt stress on genes related to photosynthesis and carbon metabolism in cotton

**Fig. 11:**
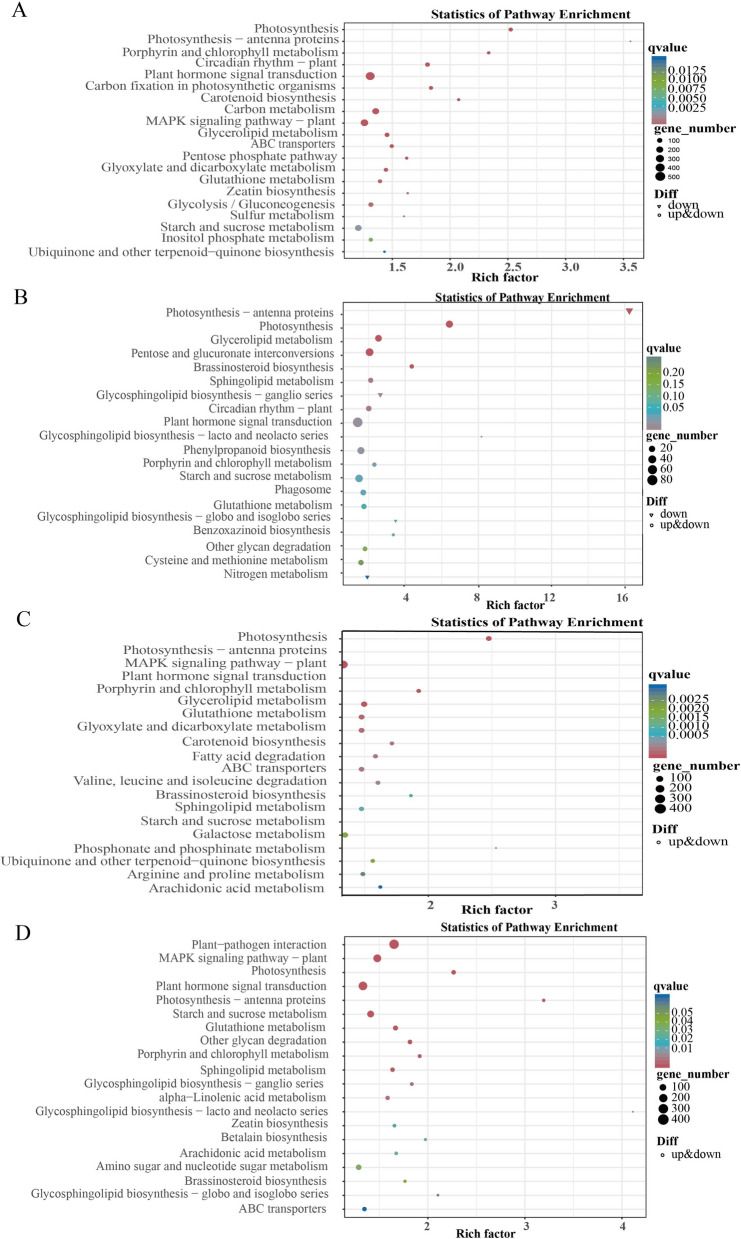
Expression of genes related to photosynthesis and carbon metabolism in cotton under salt stress and re-watering

KEGG pathway analysis revealed enrichment of"carbon fixation in photosynthetic organisms"and"photosynthesis"pathways in cotton. The expression levels of genes encoding key enzymes involved in carbon fixation in STs and SS were suppressed under salt stress (Fig. 11). Similarly, salt stress inhibited photosynthesis and reduced carbon assimilation in the ST and SS, which ultimately led to structural and functional damage to the cotton leaves. Moreover, the expression of most genes involved in photosynthesis and carbon metabolism was more affected by salt stress than by genotype, and the genes related to photosynthesis and carbon metabolism in STs and SS were suppressed under salt stress. After re-watering, more similar gene expression patterns were detected under salt stress and re-watering treatments. In particular, the expression of genes related to PSII, PSI, FNR, Fd, LHCI and LHCII in the ST decreased in response to salt stress, and after re-watering, their expression levels increased to normal levels. After re-watering, the expression levels of related genes were still downregulated after SS re-watering.

### ST improved growth hormone, gibberellin and cytokinin signaling under salt stress and re-watering to promote cotton plant growth

**Fig. 12:**
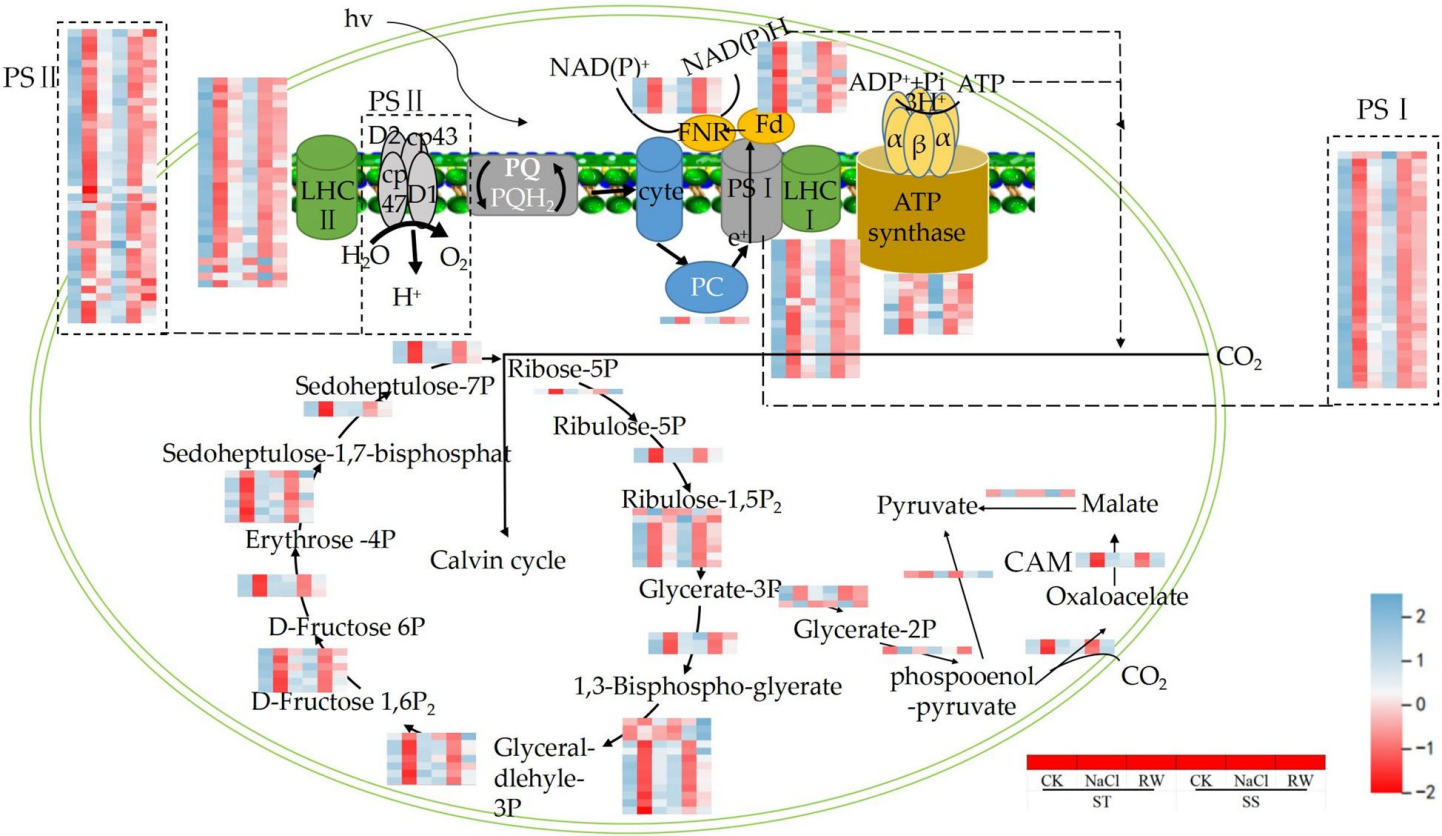
Effects of salt stress and re-watering on the expression of growth hormone-, cytokinin- and gibberellin-related genes in cotton

Growth hormones, gibberellins and cytokinins control plant growth by promoting cell elongation or division, thereby increasing salt tolerance in plants under salt stress [54]. Furthermore, the growth hormone endocytic vector (AUX1) and gibberellin receptor (GID1) are required to cope with cell damage under salt stress, and the expression of cytokinin receptors and several Arabidopsis A-type response regulators is affected by salt treatment [38, 55]. In this study, under salt stress and re-watering treatment, the expression levels of 8 genes related to AUX1 in ST47 were greater than those in SS. There were 6 genes related to GID1 whose expression levels were elevated by salt stress, and these genes showed better recovery in STs with lower expression levels after re-watering, but SS still showed higher expression levels (Fig. 12). Four genes related to A-type response regulators in ST and SS showed low expression levels under normal conditions, and 2 genes were elevated under salt stress. After re-watering, the expression levels of these 2 genes in the STs decreased and recovered. However, these 2 genes of SS were still highly expressed. Our results suggest that ST coordinately regulates growth hormone, gibberellin and cytokinin signaling and promotes improved growth and recovery under salt stress.

### Response of plant circadian genes to salt stress and re-watering in cotton

**Fig. 13:**
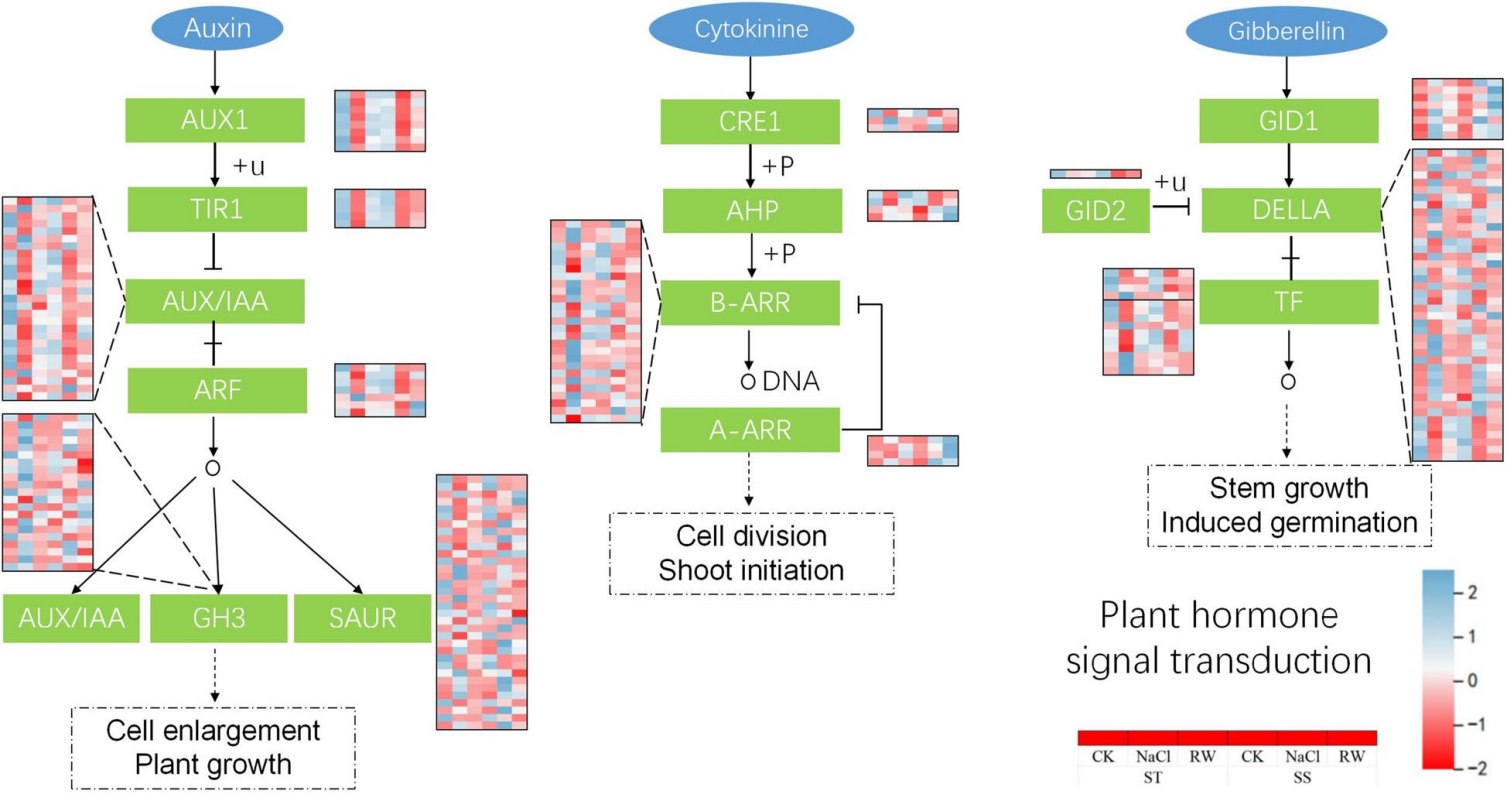
Effects of salt stress and re-watering on the expression of genes related to biological clocks in cotton

Plant circadian rhythms and their response mechanisms to various abiotic stresses have been a hot topic in plant physiology and ecology [56]. There are many different models of biological clocks. A simple model of a plant biological clock consists of three circuits: CCA1, LHY and TOC1 (PRR1), which form the core circuit; PRR5/7/9, which forms the daytime circuit; and ELF3, ELF4, GI and LUX, which form the nighttime circuit [57]. In this study, KEGG pathway analysis revealed that ST cotton was enriched in the"plant circadian rhythm"pathway under salt stress and re-watering, and two TOC1 genes in ST and SS exhibited low expression levels under normal conditions, while the expression of ST-related genes increased under salt stress and decreased after re-watering, while that of SS decreased under both salt stress and re-watering (Fig. 13). SS had lower gene expression levels in both the salt stress and re-watering treatments. Similarly, the expression of one GI and three PRR7 genes in both genotypes increased under salt stress and decreased after re-watering. There were 2 PRR9 genes in the ST, whose expression increased under salt stress and decreased after re-watering, and in the SS, whose expression decreased under both salt stress and re-watering.

### MAPK signaling in response to salt stress

**Fig. 14:**
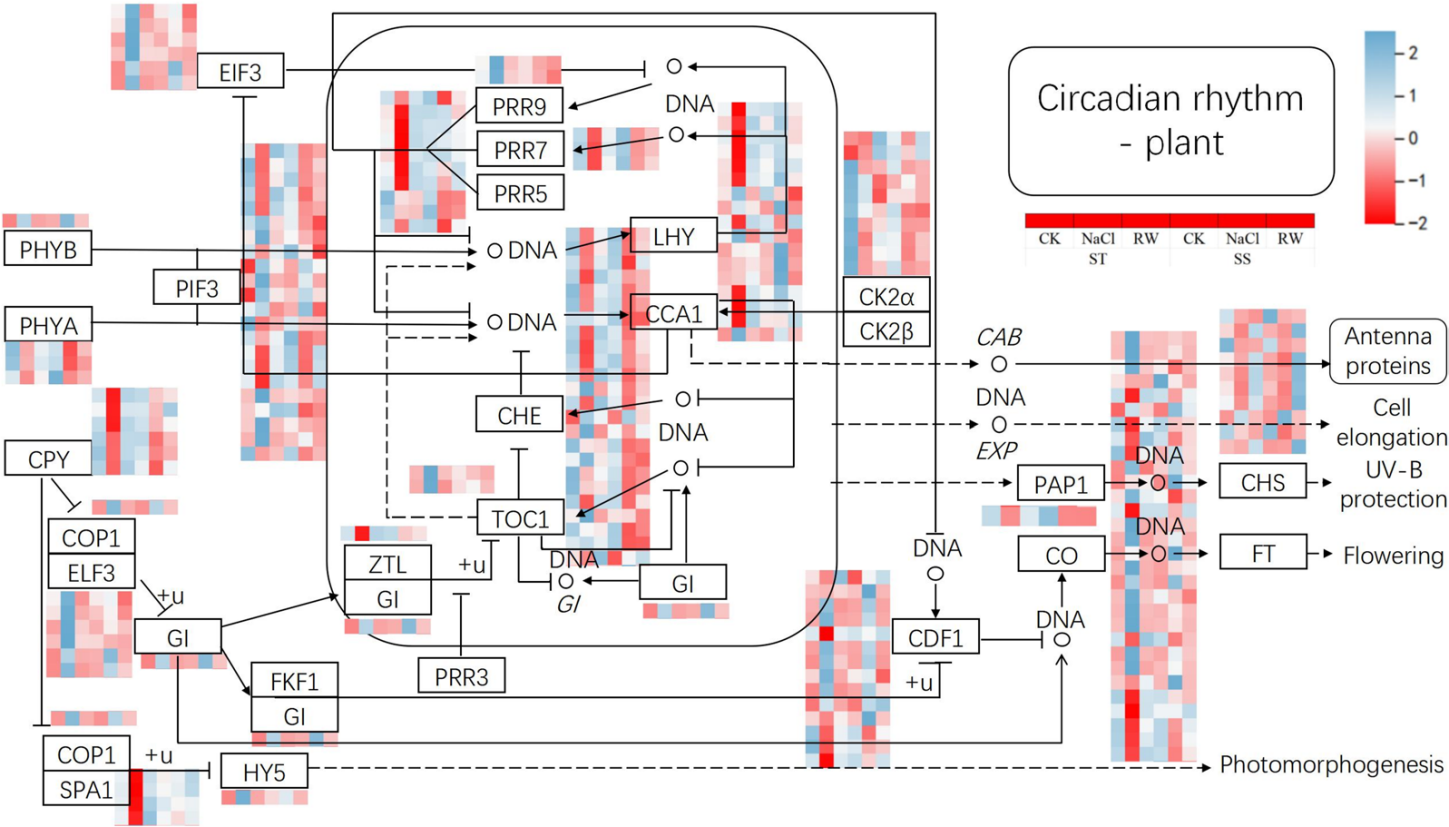
Effects of salt stress and re-watering on MAPK signal transduction-related genes in cotton

The MAPK cascade pathway plays an important role in environmental stress signaling via the MAPKKK-MAPKK-MAPK-mediated triple phosphorylation cascade to induce tolerance responses [58]. In this study, the majority of MAPK-related genes in ST and SS were altered by salt stress, and most of the genes in ST were restored to normal levels after re-watering, while most of the genes in SS were still activated to relatively high or low levels (Fig. 14). The expression of the MEKK1, MEKK2, and MEK4/6 genes in both STs and SSs was induced by salt stress, and their expression was elevated. Interestingly, after re-watering, the expression of all genes related to ST decreased, and the expression of some genes related to SS decreased, while others maintained high expression.

### WGCNA of DEGs under salt stress and re-watering

**Fig. 15:**
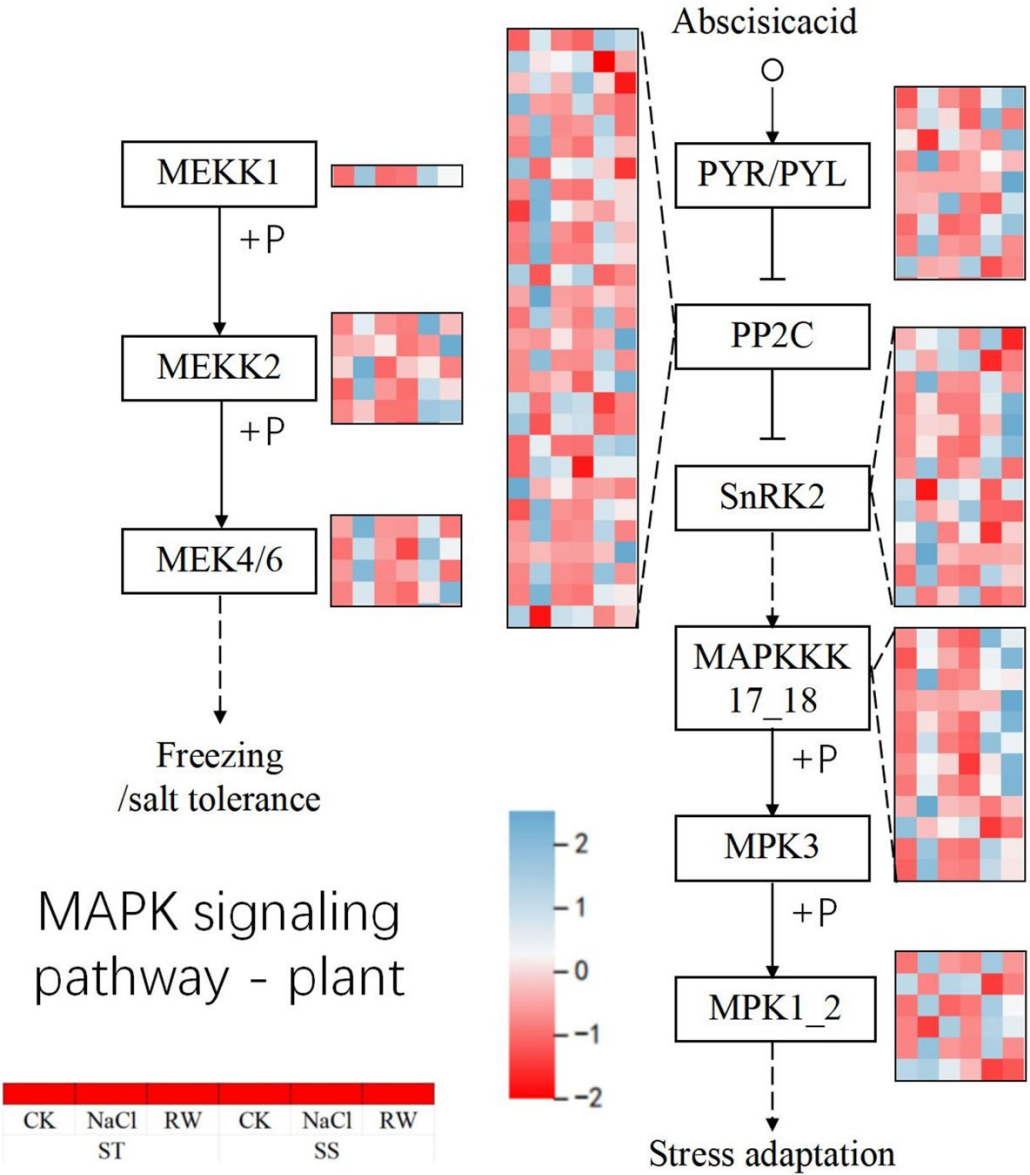
WGCNA of DEGs under salt stress and re-watering conditions. A sample cluster analysis; **B** Selection of soft threshold; **C** gene cluster tree analysis; **D** heatmap of correlation between modules and treatments

To understand gene expression and re-watering and identify candidate genes for salt tolerance, WGCNA was used to construct a coexpression network for DEGs under salt stress and re-watering (Fig. 15). The expression matrix was imported. The dist function and hclust clustering were used to calculate the sample algorithm. The graph shows no outliers among the 18 samples. There were significant differences between ST and SS under the different treatments. ST and SS can be clearly distinguished under re-watering, which implies that the expression matrix has good specificity and can be used to effectively distinguish between ST and SS under different treatments. This study selected the optimal soft threshold of 26 to construct an infinitely close scale-free network of gene coexpression. Genes were divided into six modules. The gray module had the greatest correlation with SS re-watering (R = 0.86 and *p* < 0.0001).

**Fig. 16:**
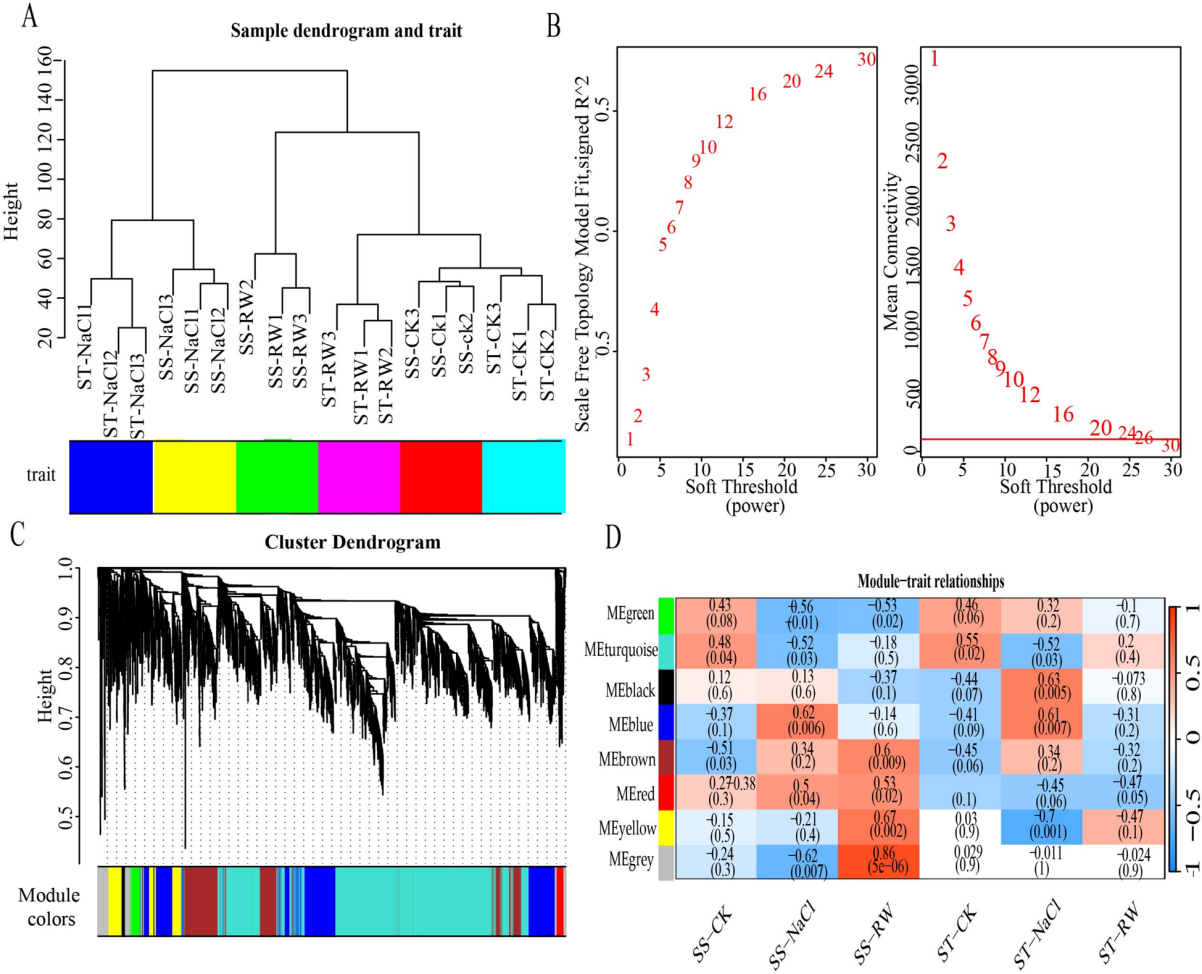
The gray module-derived network in Cytoscape was filtered with Cytohubba to extract hub genes

By using the Cytohubba plugin in Cytoscape, the top five genes with the highest correlation were selected as the hub genes for the modules (Fig. 16). Five key hubs*, namely, GH_A01G1528*, *GH_A08G2688*, *GH_D08G2683, GH_D01G1620,* and *GH*_A10G0617, were screened from among the hub genes in the MEgray module (Table 1).

**Table 1:** IDs and gene annotations of five key genes obtained from WGCNA

**Hub gene ID**	**Homologous gene ID in A.thaliana**	**Functional annotation**	**Gene name**
*GH_A01G1528*	*AT5G59310*	Nonspecific lipid-transfer protein 4	LTP4
*GH_A08G2688*	*AT5G57800*	Protein ECERIFERUM 3	CER3
*GH_D01G1620*	*AT4G38660*	Nonspecific lipid-transfer protein 1	LTP1
*GH_A10G0617*	*AT1 g17710*	Inorganic pyrophosphatase 2	PEPC1
*GH_D08G2683*	*AT5G57800*	Protein ECERIFERUM 3	CER3

## Discussion

### Response of cotton leaves to salt stress and re-watering

After 48 h of 200 mM NaCl treatment, cotton seedling leaves exhibited severe physiological and anatomical damage, marked by a significant increase in MDA concentration and reductions in chlorophyll content and SOD activity. These findings align with short-term (48–72 h) salinity stress studies on Gossypium hirsutum seedlings, where rapid lipid peroxidation and chlorophyll degradation were attributed to ROS accumulation [59]. However, contrasting patterns emerge in long-term stress experiments: salt-tolerant genotypes demonstrate delayed but sustained upregulation of antioxidant enzymes (e.g., POD and CAT) to mitigate oxidative damage [60].

Notably, our results using 200 mM NaCl differ from studies employing higher concentrations (> 200 mM). For instance, Zhao et al. reported irreversible membrane damage in cotton leaves within 24 h under 300 mM NaCl, whereas our 200 mM treatment allowed partial cellular repair post-rehydration [61]. This underscores the critical role of concentration thresholds in stress resilience. Furthermore, compared to soil-grown cotton subjected to salinity, hydroponic systems (as used here) may exacerbate Na^+^ toxicity due to unrestricted ion uptake [62]. To minimize unintended root hypoxia, we implemented foil-wrapped conical flasks and agitation—methods validated by Shabala et al. to maintain root-zone O_2_ levels > 6 mg/L in Arabidopsis hydroponics, thereby reducing confounding anaerobic stress effects [63].

Microstructural observations revealed that NaCl stress caused bulliform cells to shift from compact quadrilaterals to ovoid shapes, with loosened palisade and spongy tissue arrangements—a phenotype consistent with *G.barbadense* leaves under salinity, where cell shrinkage reduces photosynthetic efficiency [61]. Intriguingly, flowering-stage cotton plants prioritize Na^+^ exclusion via trichome secretion over leaf structural repair under comparable NaCl stress (200 mM), as demonstrated by Peng et al., highlighting developmental-stage-specific tolerance mechanisms absent in seedlings [59].

### Transcriptomic analysis of the response of cotton leaves to salt stress

ST uniquely enriched carbon fixation in photosynthetic organisms (ko00710) and carbon metabolism (ko01200) pathways under salt stress, preserving Rubisco and PEPC activities to mitigate chloroplast damage. This aligns with its smaller chlorophyll decline (− 10.29% vs. SS’s − 13.89%) and rapid post-rehydration recovery (chlorophyll at 94.77% of CK). Crucially, the sustained activation of the circadian rhythm pathway (ko04712) in ST likely regulates magnesium transporters (e.g., homologs of rice *OsMGT3*) to rhythmically activate Mg-dependent enzymes like Rubisco [64, 65]. *TOC1* (*PRR1*), a core circadian oscillator, forms a negative feedback loop with *CCA1/LHY* to fine-tune stress-responsive gene expression. In rice, *OsTOC1* overexpression enhances pathogen resistance by activating *OsTGAL3a/b* [66], suggesting a conserved role of *TOC1* in stress adaptation. This photosynthesis-circadian coupling minimizes ROS bursts, evidenced by lower MDA accumulation (+ 36.07% vs. SS’s + 64.61%), as rhythmic SOD/POD synthesis dynamically counterbalances stress-induced ROS fluctuations.

SS relied on fatty acid degradation (ko00071) and branched-chain amino acid catabolism (ko00280) to mobilize stored lipids/proteins for energy, exacerbating membrane peroxidation (MDA + 64.61%) and SOD collapse (− 27.5%). To counteract membrane dysfunction, SS activated brassinosteroid biosynthesis (ko00905) and sphingolipid metabolism (ko00600): BRs stabilize membrane integrity via lipid remodeling [67], while sphingolipids like ceramide adjust membrane fluidity under osmotic stress [68]. Notably, SS likely lacks *TOC1*-mediated rhythmic stress priming, in Arabidopsis, *TOC1* suppresses *MYB44* expression at dawn to balance cold responses with energy homeostasis [69]. SS’s circadian dysregulation may fail to timely downregulate non-essential pathways (e.g., photorespiration), exacerbating ROS accumulation. Although these compensations reduced post-rehydration MDA to + 11.54%, excessive energy expenditure left SS with 9,390 DEGs (3.8 × ST’s 2,454), delaying recovery.

Post-rehydration, ST rapidly rebuilt lipid-carbohydrate homeostasis via glycerolipid metabolism (ko00561) and pentose-glucuronate interconversion (ko00040), restoring SOD to 97.49% of CK and normalizing MDA. Phenylpropanoid biosynthesis (ko00940) likely synergized this process—lignin precursors reinforce cell walls against osmotic stress [70], while phenolic compounds directly scavenge ROS. In Arabidopsis, temperature fluctuations induce alternative splicing (AS) of *TOC1* and *PRRs*: heat stress triggers intron retention in *TOC*1, leading to its degradation and subsequent *PIF4* activation for thermomorphogenesis [71]. ST may maintain *PRR5*/*PRR7* splicing fidelity (dependent on *AtPRMT5*-mediated methylation [72]) to stabilize circadian phase, avoiding metabolic oscillations during recovery. In contrast, SS persistently activated MAPK signaling (ko04016) and α-linolenic acid metabolism (ko00592): MAPK cascades demand ATP to sustain ion transport (e.g., *SOS1*) [73, 74], explaining SS’s limited SOD recovery (89.64% of CK); JA signaling (from α-linolenic acid) induces antioxidant enzymes [75], yet SS’s POD remained 10.36% below CK, indicating repair lag.

ST’s persistent circadian rhythm pathway (ko04712) activity underpins its recovery advantage through three mechanisms, Rhythmic ion regulation: Coordinating with SOS pathway to oscillate Na⁺ transporter (e.g., *SOS1*) expression, preventing toxic accumulation; Photosynthetic-ROS synchronization, Timing PEPC/Rubisco expression to avoid photorespiration-driven ROS surges (e.g., C3 rice’s PEPC dysregulation); Antioxidant enzyme phasing, Aligning SOD/POD synthesis peaks with ROS generation periods. The *TOC1*-*PRR*-*CCA1*/*LHY* feedback loop is central to this process, in Arabidopsis, *PRR9/PRR7* mutations cause aberrant activation of cold-induced *DREB1* [76], while ST likely fine-tunes this loop to prevent overactivation of stress-responsive genes (e.g., *DREB1*), maintaining energy homeostasis. This “predictive regulation” enables ST to swiftly repair chloroplasts post-rehydration, while SS’s circadian decoupling prolongs metabolic dysregulation (9,390 DEGs), hindering recovery.

ST's salt tolerance hinges on circadian-regulated cellular processes (e.g., rhythmic Na⁺/ROS control via *TOC1*-*SOS1* coordination) and stress-adaptive molecular binding (e.g., Mg^2^⁺-dependent Rubisco activation), while SS's reliance on energy-draining lipid catabolism exacerbates oxidative damage [64–75].

### Molecular mechanisms of the salt stress response in cotton

Salt stress can cause multiple injuries to plants, such as osmotic stress, ionic toxicity, and oxidative damage, which in turn reduce photosynthesis and inhibit plant growth and development processes [77]. Oxidative damage, mediated by excessive ROS accumulation, is a critical component of salt stress, as ROS disrupt cellular homeostasis by damaging lipids, proteins, and nucleic acids. Plants have evolved sophisticated mechanisms to maintain ROS homeostasis, including enzymatic and non-enzymatic antioxidant systems, which are crucial for salt tolerance [78].

In salt-stressed plants, ROS such as superoxide anion (O^₂^⁻) and hydrogen peroxide (H₂O₂) are primarily generated in chloroplasts, mitochondria, and peroxisomes. The balance between ROS production and scavenging is tightly regulated by antioxidant enzymes, including SOD, CAT, APX, and GPX [79]. In this study, ST and SS exhibited significant differences in ROS management: ST maintained lower MDA levels (+ 36.07% vs. SS’s + 64.61%), indicating less membrane lipid peroxidation, likely due to higher SOD and POD activities (ST: − 3.06%/− 12.98% vs. SS: − 27.5%/− 13.89%). SS’s antioxidant system was severely compromised, as evidenced by its delayed recovery of SOD activity (89.64% of CK) and elevated MDA levels (+ 11.54%) after re-watering.

Comparative studies in rice and Arabidopsis highlight the importance of ROS scavenging in salt tolerance. For instance, the *EGY3* gene in Arabidopsis stabilizes Cu/Zn-SOD2 (CSD2) in chloroplasts, enhancing H₂O₂ production and retrograde signaling to upregulate nuclear-encoded stress-responsive genes [80]. Similarly, in soybean, the transcription factor *GmNTL1* undergoes H₂O₂-dependent oxidation, promoting ROS signaling and activating Na⁺/H⁺ antiporters (e.g., *GmNHX1*) to maintain ion homeostasis [81]. These findings suggest that enhancing ROS scavenging capacity, particularly in chloroplasts, could improve salt tolerance in cotton.

Plant growth and development require low levels of Na⁺, and excessive accumulation can disrupt ionic balance, leading to physiological and metabolic disorders [82]. The SOS pathway plays a central role in Na⁺ exclusion, involving SOS1 (Na⁺/H⁺ antiporter), SOS2 (protein kinase), SOS3 (calcium-binding protein), and SCaBP8. Under salt stress, calcium (Ca^2^⁺) signals activate SOS3, which recruits and phosphorylates SOS2, ultimately activating SOS1 to extrude Na⁺ from cells [83]. In this study, both ST and SS enriched DEGs related to the SOS pathway, but ST exhibited better recovery of ion homeostasis after re-watering, likely due to its more efficient Ca^2^⁺ signaling and SOS pathway activation. Interestingly, the circadian clock gene *GI* (*GIGANTEA*) interacts with SOS2, modulating its activity and enhancing Na⁺ exclusion in Arabidopsis. This suggests that ST’s robust circadian regulation may contribute to its superior ion homeostasis under salt stress.

Osmotic stress reduces cell expansion, affects water use efficiency, and induces stomatal closure, disrupting photosynthetic pigments [84]. In this study, both ST and SS showed significant repression of photosynthesis-related genes (ko00195, ko00196) under salt stress, but ST maintained higher expression levels, correlating with its smaller chlorophyll decline (− 10.29% vs. SS’s − 13.89%) and faster recovery after re-watering (chlorophyll at 94.77% of CK). This aligns with findings in rice, where potassium (K⁺) application upregulates antioxidant enzymes (e.g., SOD, APX) and enhances photosynthetic recovery under salt stress [85].

ST’s enrichment in carbon fixation (ko00710) and carbon metabolism (ko01200) pathways further supports its ability to sustain photosynthetic efficiency and energy production under stress. In contrast, SS relied on fatty acid and amino acid degradation (ko00071, ko00280), a less efficient energy-generating strategy that exacerbates ROS accumulation and delays recovery. Phytohormones such as ABA and ethylene play pivotal roles in salt stress responses. ABA enhances stomatal closure and activates stress-responsive genes, while ethylene negatively regulates salt tolerance by promoting ROS accumulation and disrupting ion homeostasis [86]. In this study, DEGs related to phytohormone signaling and MAPK pathways were enriched in both ST and SS, but ST exhibited better recovery, suggesting more efficient hormone-mediated stress adaptation.For example, ABA signaling activates SnRK2 kinases, which phosphorylate transcription factors like AREB/ABF to regulate ion homeostasis and ROS scavenging. In contrast, ethylene signaling via *OsSIT1* in rice promotes ROS accumulation and Na⁺ sensitivity, highlighting the need to balance hormone signaling for optimal stress tolerance [87].

### Identification of salt tolerance-related coexpression modules in cotton leaves via WGCNA

WGCNA is a bioinformatics analysis method based on large amounts of transcriptome sample data. It assumes that the gene network follows a scale-free distribution, clusters genes with similar expression patterns into different modules, analyzes correlations between the modules and specific traits or phenotypes, and constructs a coexpression regulatory network based on gene clustering and association analysis results. Genes located at the center of the regulatory network are referred to as hub genes, which are usually key regulatory genes and are worthy of in-depth exploration and analysis [88].

Our study used WGCNA to enrich a gray coexpression module associated with cotton restoration under salt stress. By screening the gray module, several key genes were identified. Notably, lipid transfer proteins (LTPs) exhibit functional diversity in stress adaptation: maize and *Brassica napus* pan-genome studies reveal their involvement in male fertility and developmental regulation, while soybean *GmLtpI.3* enhances drought/salt tolerance through ROS signaling and osmotic adjustment [89–91]. *GH_A01G1528* (LTP4, lipid transfer protein) and *GH_D01G1620* (LTP1, lipid transfer protein) are essential active proteins involved in plant life activities, accounting for 4% of cell soluble proteins [92]. In xerophytes like *Ammopiptanthus mongolicus*, *LTPs* (e.g., *GH_A08G2688*) coordinate with ABC transporters to deliver cuticular wax under PEG-induced drought, while cotton *GhMYB4* transcriptionally suppresses *GhLTP4* to regulate fiber elongation via lipid-sucrose signaling [91, 93]. *LTPs* can respond to low temperature, drought, salt stress, infections by bacterial and fungal pathogens, and environmental changes in signaling molecules, such as abscisic acid (ABA), salicylic acid (SA), and ethylene [94–96]. Combining transcription analysis of *Arabidopsis* wild-type and tdr1 mutant plants under NaCl treatment with yeast one-hybrid experiments, it can be seen that LTP4 may serve as a direct target gene for *TDR1*, mediating the regulation of *TDR1* in plant salt tolerance [97]. As a positive regulatory factor, *NtLTP4* participates in the response of plants to abiotic stresses. Under high salt and drought stress conditions, compared with those in the wild-type control group, the *NtLTP4-*overexpressing strains exhibited extremely strong tolerance, faster seed germination, longer seedling roots, and slower water loss from leaves [98, 99]. *GH_A08G2688* (CER3) is an important gene involved in the biosynthesis of long-chain alkanes [100–102]. In *Brassica napus*, CER3 homologs show dynamic expression under phytohormone treatments, with significant downregulation by MeJA and ACC [102]. *GH_A10G0617* (PEPC1) is an important enzyme in plants that regulates carbon flow through the TCA cycle and controls protein and oil biosynthesis [103]. Comparative studies reveal species-specific PEPC functions: C4 foxtail millet maintains stable *SiPEPC4* expression under salt stress, whereas C3 rice exhibits PEPC upregulation potentially exacerbating photorespiration-derived ROS. Silicon nanoparticles mitigate salt-low temperature dual stress by protecting PEPC/Rubisco activity, thereby reducing photosynthetic ROS burst [104, 105]. The expression of *PEPC1* (1 A and 1D) in cotton is induced by cold and salt stresses [106]. Overall, these genes may play a critical role in the response of plants to salt stress. Intriguingly, cassava leverages PEPC-mediated CO₂ recycling to enhance leaf water-use efficiency under drought, suggesting evolutionary conservation of PEPC in metabolic adaptation Overall, these genes may play a critical role in the response of plants to salt stress [107]. However, further research is needed to reveal their specific mechanisms of action.

## Conclusion

In summary, the present study systematically analyzed the differences in the response of different salt-tolerant cotton plants to salt stress at the physiological, biochemical, microstructural and transcriptomic levels. Under salt stress, the cotton malondialdehyde content increased, and the chlorophyll content, superoxide dismutase activity and peroxidase activity decreased; after re-watering, both cotton genotypes recovered somewhat, and ST performed significantly better than did SS. Similarly, leaf microstructural observations revealed that salt stress induced structural changes in cells and tissues; after re-watering, the STs exhibited improved recovery. By comparing the transcriptome data of ST and SS, 15,780/14156 and 2454/9390 DEGs related to salt tolerance were identified at 48 h NaCl treatment and 48 h RW treatment, respectively. GO and KEGG pathway enrichment analyses revealed a cultivar of organismal responses to salt stress, including photosynthesis (ko 00195), photosynthesis-antenna protein (ko 00196), plant hormone signal transduction (ko 04075), starch and sucrose metabolism (ko 00500), and porphyrin and chlorophyll metabolism (ko 00860), among other interactions. WGCNA revealed enriched gray coexpression modules related to the recovery of cotton plants under salt stress, and screening of the pivotal genes in the gray module revealed five critical hubs, namely, *GH_A01G1528*, *GH_A08G2688*, *GH_D08G2683*, *GH_D01G1620* and *GH_A10G0617*.

## Supplementary Information

Supplementary Material 1: Figure S1. Relationships between 18 samples.

Supplementary Material 2: Table S1. Growth traits and salt injury rates of different cotton genotypes under salt stress. Table S2. Names, annotations, and primers of randomly selected DEGs. Table S3. Epidermal structure of cotton seedling leaves under salt stress and re-watering. Table S4. Summary of RNA-Seq results under salt stress and re-watering conditions.

## Data Availability

RNA-seq raw data were also deposited under these NCBI accessions of PRJNA1133963.
