# Supplementary material for: Response of different cotton genotypes to salt stress and re-watering
Source: BMC Plant Biol. 2025 May 5;25:587. doi: 10.1186/s12870-025-06534-6 (PMC12051324; doi:10.1186/s12870-025-06534-6)
Supplement: Supplementary file 1 — Supplementary Material 1: Figure S1. Relationships between 18 samples. [file 12870_2025_6534_MOESM1_ESM.docx]

**Supplementary figure**

**
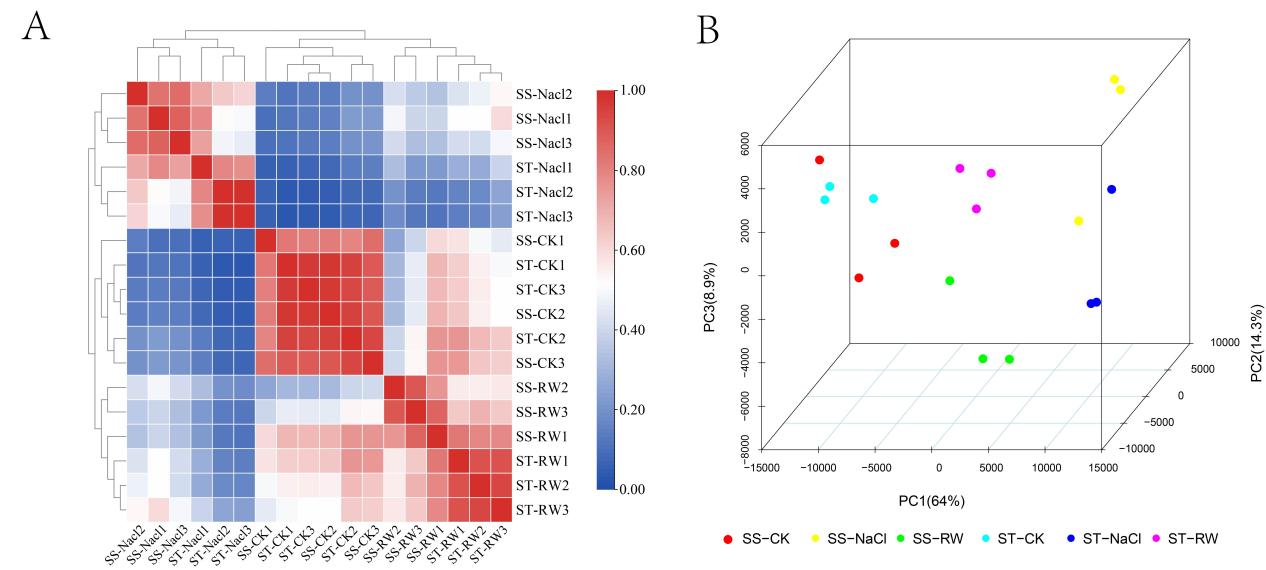
**

**Fig.S1 Relationship between 18 samples.**

1. Cluster dendrogram and Pearson correlation coefficient heatmap based on standardized FPKM values of expressed genes. The abscissa and ordinate in the graph are sample numbers, and their order is determined by the results of sample correlation clustering. The top and left sides of the graph are corresponding cluster trees, and the color reflects the correlation degree between samples. (B)Principal component analysis of identified genes

**Fig.S2 Relationship between RNA-seq and quantitative real-time PCR（qPCR）expression data（log2 fold change)(R^2^=0.80)**
